# Mango pangenome reveals dramatic impacts of reference bias on population genomic analyses

**DOI:** 10.1093/hr/uhaf166

**Published:** 2025-07-01

**Authors:** Bilal Ahmad, Ying Su, Yani Hao, Tayyaba Razzaq, Rida Arshad, Yi Zhang, Yingchun Zhang, Xingyi Wang, Guizhou Huang, Xiangnian Su, Ting Hou, Chaochao Li, Xuanwen Yang, Chuanning Li, Zhenzhou Chu, Qiuyan Wang, Yu Zhang, Zhongxin Jin, Qi Xu, Xiaodong Xu, Yanling Peng, Guiqi Bi, Chengjie Chen, Yeyuan Chen, Hua Xiao, Jianfeng Huang, Yongfeng Zhou, Xinmin Tian

**Affiliations:** State Key Laboratory of Tropical Crop Breeding, Shenzhen Branch, Guangdong Laboratory of Lingnan Modern Agriculture, Key Laboratory of Synthetic Biology, Ministry of Agriculture and Rural Affairs, Agricultural Genomics Institute at Shenzhen, Chinese Academy of Agricultural Sciences 518000, Shenzhen, China; State Key Laboratory of Tropical Crop Breeding, Shenzhen Branch, Guangdong Laboratory of Lingnan Modern Agriculture, Key Laboratory of Synthetic Biology, Ministry of Agriculture and Rural Affairs, Agricultural Genomics Institute at Shenzhen, Chinese Academy of Agricultural Sciences 518000, Shenzhen, China; Xinjiang Key Laboratory of Biological Resources and Genetic Engineering, College of Life Science and Technology, Xinjiang University, Urumqi, Xinjiang 830046, China; State Key Laboratory of Tropical Crop Breeding, Shenzhen Branch, Guangdong Laboratory of Lingnan Modern Agriculture, Key Laboratory of Synthetic Biology, Ministry of Agriculture and Rural Affairs, Agricultural Genomics Institute at Shenzhen, Chinese Academy of Agricultural Sciences 518000, Shenzhen, China; Department of Horticulture, Hainan Institute of Northwest A&F University, Sanya 572024, China; State Key Laboratory of Tropical Crop Breeding, Shenzhen Branch, Guangdong Laboratory of Lingnan Modern Agriculture, Key Laboratory of Synthetic Biology, Ministry of Agriculture and Rural Affairs, Agricultural Genomics Institute at Shenzhen, Chinese Academy of Agricultural Sciences 518000, Shenzhen, China; State Key Laboratory of Tropical Crop Breeding, Shenzhen Branch, Guangdong Laboratory of Lingnan Modern Agriculture, Key Laboratory of Synthetic Biology, Ministry of Agriculture and Rural Affairs, Agricultural Genomics Institute at Shenzhen, Chinese Academy of Agricultural Sciences 518000, Shenzhen, China; State Key Laboratory of Tropical Crop Breeding, Shenzhen Branch, Guangdong Laboratory of Lingnan Modern Agriculture, Key Laboratory of Synthetic Biology, Ministry of Agriculture and Rural Affairs, Agricultural Genomics Institute at Shenzhen, Chinese Academy of Agricultural Sciences 518000, Shenzhen, China; State Key Laboratory of Tropical Crop Breeding, Shenzhen Branch, Guangdong Laboratory of Lingnan Modern Agriculture, Key Laboratory of Synthetic Biology, Ministry of Agriculture and Rural Affairs, Agricultural Genomics Institute at Shenzhen, Chinese Academy of Agricultural Sciences 518000, Shenzhen, China; State Key Laboratory of Tropical Crop Breeding, Shenzhen Branch, Guangdong Laboratory of Lingnan Modern Agriculture, Key Laboratory of Synthetic Biology, Ministry of Agriculture and Rural Affairs, Agricultural Genomics Institute at Shenzhen, Chinese Academy of Agricultural Sciences 518000, Shenzhen, China; College of Forestry, Beijing Forestry University, 100083 Beijing, China; State Key Laboratory of Tropical Crop Breeding, Shenzhen Branch, Guangdong Laboratory of Lingnan Modern Agriculture, Key Laboratory of Synthetic Biology, Ministry of Agriculture and Rural Affairs, Agricultural Genomics Institute at Shenzhen, Chinese Academy of Agricultural Sciences 518000, Shenzhen, China; State Key Laboratory of Tropical Crop Breeding, Shenzhen Branch, Guangdong Laboratory of Lingnan Modern Agriculture, Key Laboratory of Synthetic Biology, Ministry of Agriculture and Rural Affairs, Agricultural Genomics Institute at Shenzhen, Chinese Academy of Agricultural Sciences 518000, Shenzhen, China; State Key Laboratory of Tropical Crop Breeding, Shenzhen Branch, Guangdong Laboratory of Lingnan Modern Agriculture, Key Laboratory of Synthetic Biology, Ministry of Agriculture and Rural Affairs, Agricultural Genomics Institute at Shenzhen, Chinese Academy of Agricultural Sciences 518000, Shenzhen, China; State Key Laboratory of Tropical Crop Breeding, Tropical Crops Genetic Resources Institute, Chinese Academy of Tropical Agricultural Sciences, Haikou 571100, China; State Key Laboratory of Tropical Crop Breeding, Shenzhen Branch, Guangdong Laboratory of Lingnan Modern Agriculture, Key Laboratory of Synthetic Biology, Ministry of Agriculture and Rural Affairs, Agricultural Genomics Institute at Shenzhen, Chinese Academy of Agricultural Sciences 518000, Shenzhen, China; Key Laboratory of Ecology of Rare and Endangered Species and Environmental Protection (Ministry of Education) & Guangxi Key Laboratory of Landscape Resources Conservation and Sustainable Utilization in Lijiang River Basin, Guangxi University Engineering Research Center of Bioinformation and Genetic Improvement of Specialty Crops, Guangxi 541006, China; Xinjiang Key Laboratory of Biological Resources and Genetic Engineering, College of Life Science and Technology, Xinjiang University, Urumqi, Xinjiang 830046, China; Key Laboratory of Ecology of Rare and Endangered Species and Environmental Protection (Ministry of Education) & Guangxi Key Laboratory of Landscape Resources Conservation and Sustainable Utilization in Lijiang River Basin, Guangxi University Engineering Research Center of Bioinformation and Genetic Improvement of Specialty Crops, Guangxi 541006, China; Key Laboratory of Ecology of Rare and Endangered Species and Environmental Protection (Ministry of Education) & Guangxi Key Laboratory of Landscape Resources Conservation and Sustainable Utilization in Lijiang River Basin, Guangxi University Engineering Research Center of Bioinformation and Genetic Improvement of Specialty Crops, Guangxi 541006, China; Guangxi Subtropical Research Institute, Guangxi Academy of Agricultural Sciences, Nanning 530001,China; State Key Laboratory of Tropical Crop Breeding, Tropical Crops Genetic Resources Institute, Chinese Academy of Tropical Agricultural Sciences, Haikou 571100, China; State Key Laboratory of Tropical Crop Breeding, Shenzhen Branch, Guangdong Laboratory of Lingnan Modern Agriculture, Key Laboratory of Synthetic Biology, Ministry of Agriculture and Rural Affairs, Agricultural Genomics Institute at Shenzhen, Chinese Academy of Agricultural Sciences 518000, Shenzhen, China; State Key Laboratory of Tropical Crop Breeding, Shenzhen Branch, Guangdong Laboratory of Lingnan Modern Agriculture, Key Laboratory of Synthetic Biology, Ministry of Agriculture and Rural Affairs, Agricultural Genomics Institute at Shenzhen, Chinese Academy of Agricultural Sciences 518000, Shenzhen, China; State Key Laboratory of Tropical Crop Breeding, Tropical Crops Genetic Resources Institute, Chinese Academy of Tropical Agricultural Sciences, Haikou 571100, China; State Key Laboratory of Tropical Crop Breeding, Shenzhen Branch, Guangdong Laboratory of Lingnan Modern Agriculture, Key Laboratory of Synthetic Biology, Ministry of Agriculture and Rural Affairs, Agricultural Genomics Institute at Shenzhen, Chinese Academy of Agricultural Sciences 518000, Shenzhen, China; State Key Laboratory of Tropical Crop Breeding, Tropical Crops Genetic Resources Institute, Chinese Academy of Tropical Agricultural Sciences, Haikou 571100, China; State Key Laboratory of Tropical Crop Breeding, Tropical Crops Genetic Resources Institute, Chinese Academy of Tropical Agricultural Sciences, Haikou 571100, China; Sanya Research Institute of Chinese Academy of Tropical Agricultural Sciences, Sanya 572025, China; State Key Laboratory of Tropical Crop Breeding, Shenzhen Branch, Guangdong Laboratory of Lingnan Modern Agriculture, Key Laboratory of Synthetic Biology, Ministry of Agriculture and Rural Affairs, Agricultural Genomics Institute at Shenzhen, Chinese Academy of Agricultural Sciences 518000, Shenzhen, China; State Key Laboratory of Tropical Crop Breeding, Tropical Crops Genetic Resources Institute, Chinese Academy of Tropical Agricultural Sciences, Haikou 571100, China; State Key Laboratory of Tropical Crop Breeding, Shenzhen Branch, Guangdong Laboratory of Lingnan Modern Agriculture, Key Laboratory of Synthetic Biology, Ministry of Agriculture and Rural Affairs, Agricultural Genomics Institute at Shenzhen, Chinese Academy of Agricultural Sciences 518000, Shenzhen, China; State Key Laboratory of Tropical Crop Breeding, Tropical Crops Genetic Resources Institute, Chinese Academy of Tropical Agricultural Sciences, Haikou 571100, China; Xinjiang Key Laboratory of Biological Resources and Genetic Engineering, College of Life Science and Technology, Xinjiang University, Urumqi, Xinjiang 830046, China; Key Laboratory of Ecology of Rare and Endangered Species and Environmental Protection (Ministry of Education) & Guangxi Key Laboratory of Landscape Resources Conservation and Sustainable Utilization in Lijiang River Basin, Guangxi University Engineering Research Center of Bioinformation and Genetic Improvement of Specialty Crops, Guangxi 541006, China

## Abstract

Most genomic studies start by mapping sequencing data to a reference genome. The quality of reference genome assembly, genetic relatedness to the studied population, and the mapping method employed directly impact variant calling accuracy and subsequent genomic analyses, introducing reference bias and resulting in erroneous conclusions. However, the impacts of reference bias have gained limited attention. This study compared population genomic analyses using four different reference genomes of mango (*Mangifera indica*), including the two haploid assemblies of haplotype-resolved telomere-to-telomere (T2T) genome assembly, a pangenome, and an older version of the reference genome available on NCBI. The choice of reference genome dramatically impacted the mapping efficiency and resulted in notable differences in calling the genetic variants, particularly structural variations (SVs). Phylogenetic analysis was more sensitive to the reference genome compared to genetic differentiation. Population genomic analyses of artificial selection in domestication and SV hotspot regions varied across reference genomes. Notably, the gene enrichment analyses showed significant differences in the top enriched biological processes depending on the reference genome used. Overall, the mango pangenome outperformed the other reference genomes across various metrics, followed by T2T reference genomes, as they captured greater diversity and effectively reduced reference bias. Our findings highlight the role of the mango pangenome in reducing reference bias and underscore the critical role of reference genome selection, suggesting that it is one of the most important factors in population genomic studies.

## Introduction

The reference genome assembly is the foundation for all genomic data and databases. It directly influences crucial steps in genomic analysis workflows, including genome assembly, variant identification, sequence alignment, gene annotation, and functional analysis. Genes are identified by their loci, with positions determined based on reference genome coordinates. Variants and alleles are classified by comparing them to the reference genome (i.e. reference (REF) vs. alternative (ALT)), while diploid and individual genomes are assembled using the reference genome as a template [1]. This central role in numerous genomic workflows rationalizes the need for quality reference genomes for accurate and comprehensive genomic studies. The quality of the genome assembly and the genetic similarity between the reference and the target samples substantially affect the mapping rate, downstream analyses, and accuracy of derived inferences [2, 3].

Poorly assembled or fragmented reference genomes can impede genomic analyses and introduce reference bias, especially in complex genomic regions such as centromeric and repetitive sequences [4, 5]. The primary grape genome assembly (12X.v0) lacked 9018 genes and contained 9429 gaps [6, 7] leading to inaccuracies (reference bias) in variant estimation. For example, if reference alleles are rare, variant callers may incorrectly identify more “variants,” or miss rare variants shared with the reference genome [8, 9]. The use of high-quality, gapless telomere-to-telomere (T2T) reference genomes can improve variant calling reliability and reduce bias by providing more complete data on complex regions. However, T2T genomes cannot fully eliminate reference bias, as a single genome cannot capture the full genetic diversity of a species, such as population-specific loci [10, 11]. Moreover, the genetic similarity between the reference and target samples significantly influences the mapping rate, impacting downstream analysis and interpretations. Closely related samples tend to achieve higher mapping rates, while distantly related ones often exhibit lower mapping rates, suggesting reference bias—differential mapping tendencies [2, 3]. Studies, such as those on three-spined sticklebacks, suggested that local reference genomes can reduce reference bias [12]. These studies highlight how the choice of a reference genome can influence the results of population genomic analyses.

Another key focus is the impact of reference genomes on population genomic variation analysis, population history dynamics, and genotype–phenotype association studies. Many studies of complex traits and diseases rely on the accurate identification of single nucleotide variants, small insertions/deletions, and structural variants (SVs) [13]. When evaluating reference genomes, it is crucial to consider their overall quality and representativeness. A pangenome allows a more detailed analysis of the species' genetic diversity [14]. Therefore, the most common recommendation to reduce reference bias is using pangenome rather than relying solely on single or multiple reference genomes. In population analyses, using a pangenome, rather than a single reference genome, can yield significantly different and more detailed insights. This approach allows the identification of the entire spectrum of genomic diversity, unique genes, and variants within the population. Moreover, this holds the potential to give an in-depth understanding of genetic variation and its impact on phenotypic diversity. Therefore, pangenome as a reference can be more effective in exploring the genetic basis of complex traits [15, 16]. However, no studies have been performed on highly heterozygous genomic materials to compare the impact of different genomes on population genomic studies.

In this study, we used mango as a model plant to explore the impact of different reference genomes, including a pangenome, on the downstream analysis of genomic data. Mango, owing to its superior taste, flavor, and physical appearance known as the “king of fruits,” is the world's second most important tropical fruit crop that is being grown in all continents except Antarctica. It is diploid (2*n* = 40) and belongs to the genus *Mangifera* of the Anacardiaceae family and is characterized by high heterozygosity due to cross-pollination. There are 69 species in the *Mangifera* genus, and 26 are edible [17–19]. Comparatively scant research has been done to improve the mango crop compared to other fruit crops like grapes, citrus, apples, etc. This might be attributed to the lack of a high-quality reference genome and can be the primary reason for the slow progress in mango crop improvement. Assembling the mango genome has remained challenging due to high heterozygosity [20]. After several attempts, the first chromosome-scale genome assembly of the mango cultivar “Alphonso” was developed in 2020 [21]. Subsequently, genome assemblies for other mango cultivars (“Tomy Atkins,” “Hong Xiang Ya,” and “Irwin”) and land race (San Nian Mang) have been reported [17, 22–24]. Moreover, a high-quality assembly of “Irwin” has recently been constructed using HiFi reads. The assembly has a coverage of 204× and spans 365 megabases (Mb), achieving a near-gapless chromosome-level assembly [25]. This has high contiguity, completeness, and correctness but encompasses three gaps. Although different chromosome-level mango assemblies have been generated, a gapless T2T assembly is still lacking.

We generated a high-quality, gapless, haplotype-resolved T2T genome assembly for the mango cultivar “Alphonso,” which served as the foundation for constructing a comprehensive pangenome that integrates three previously published mango genomes. To assess the influence of reference genome selection on population genomics, we compared four reference genomes: (i) CATAS_Mindica_2.1 (the old reference genome, hereafter referred to as Mindica_2.1), (ii) ALT2T_HAP1, (iii) ALT2T_HAP2, and (iv) pangenome for variant calling and subsequent downstream analysis. Our analyses evaluated how different reference genomes affect variant calling and subsequent downstream population genomic inferences. Our findings underpin the substantial impact of mango pangenome on mapping efficiency, variant detection, and data interpretation, with potential implications for genomic studies.

## Results

### Haplotype-resolved T2T gap-free reference genome for mango

**Figure 1:**
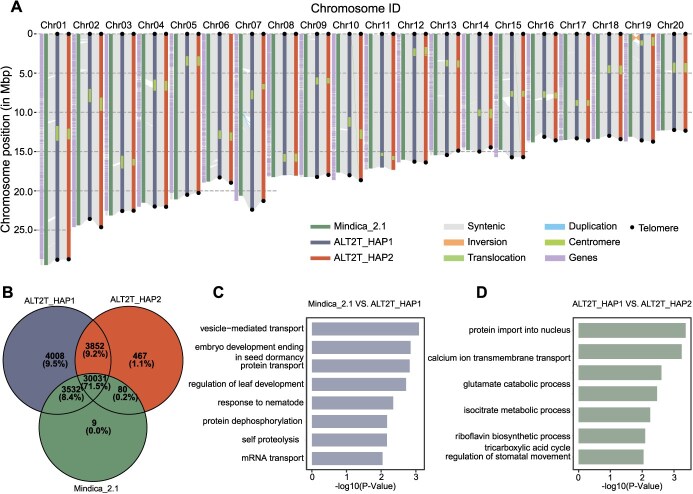
The haplotype-resolved T2T genome assembly of mango. (A) Comparative genomic analysis of three assemblies (Mindica_2.1, ALT2T_HAP1, and ALT2T_HAP2). (B) Venn diagram displaying the unique and shared genes among the three genome assemblies. (C) GO enrichment analysis of genes with insertions in the ALT2T_HAP1 genome compared to Mindica_2.1. (D) GO enrichment analysis of genes with insertions in the ALT2T_HAP2 genome compared to ALT2T_HAP1

The renowned monoembryonic Indian cultivar “Alphonso” was used to generate a diploid T2T genome assembly. To achieve this, 27.3 Gb of HiFi reads (~88.4× coverage), 44.13 Gb of ONT reads (~149.4× coverage) were combined, and 23.96 Gb of Hi-C reads were used for chromosome ordering and scaffolding. The reads were assembled using HiFiasm (v0.16) to produce a contig-level assembly. After the initial assembly, three gaps were identified—two in ALT2T_HAP1 and one in ALT2T_HAP2. Scaffolding was performed using Juicer and 3D-DNA, followed by manual gap filling. This resulted in a haplotype-resolved, gapless mango genome assembly, with sizes of 346.22 and 347.59 Mb, named ALT2T_HAP1 and ALT2T_HAP2, respectively (Fig. 1A). ALT2T_HAP1 and ALT2T_HAP2 chromosomes showed strong consistency in the Hi-C contact matrix, confirming their order and orientation correctness (Fig. S1). The genome's homozygosity and completeness, determined using *k*-mer metrics and Benchmarking Universal Single-Copy Orthologs (BUSCO), were 98.7% and 99.1%, respectively (Figs S2 and S3). Telomeres were identified at both ends of seventeen chromosomes, while only one end was identified for chromosomes 8, 11, and 19. Additionally, centromeres were identified in all 20 chromosomes (Table S1; Fig. S4). Orientation errors in Mindica_2.1 assembly were also corrected, for example, large inversion at the start of chromosome 19 (Fig. 1A).

The average hamming error and switch error rates were 1.02% and 0.014%, respectively, suggesting high phasing accuracy. Furthermore, > 99% mapping rate of HiFi reads endorses the high genome accuracy. The present genome indicates high quality over the Mindica_2.1 and all available genomes in several metrics: greater continuity (gap-free), higher completeness, larger N50 contig length (17.03 Mb), and the identification of telomeres and centromeres. The availability of high-quality gap-free assembly of mango will facilitate genomic research. The detailed comparative genomic analysis of all available genomes is presented in Table S2.

The genome assemblies ALT2T_HAP1 and ALT2T_HAP2 contained 41 423 and 40 794 protein-coding genes, respectively. A total of 4008 genes were specific to ALT2T_HAP1, while 467 and 9 genes were specific to ALT2T_HAP2 and Mindica_2.1, respectively. The presence of more unique genes in ALT2T assemblies indicates higher genome sequence completeness and increased accuracy than Mindica_2.1 (Fig. 1B). ALT2T_HAP1 and ALT2T_HAP2 were revealed to have 291 342 and 290 264 transposable elements (TEs), making up 37.05% and 37.71% of the two genomes, respectively. The two most prevalent types of TE were terminal inverted repeats (TIRs; 14.51% in HAP1, 13.18% in HAP2) and long-terminal repeat retrotransposons (LTR-RTs; 15.12% in HAP1, 16.72% in HAP2) (Fig. S5). Gene ontology (GO) enrichment analysis suggested that genes specific to ALT2T_HAP1 and ALT2T_HAP2 assemblies, compared with Mindica_2.1, are linked with important biological processes (BPs) such as seed dormancy, protein transport, regulation of leaf development, and response to nematodes (Fig. 1C and D).

### A comparison of variant calling efficiency using different reference genomes

**Figure 2:**
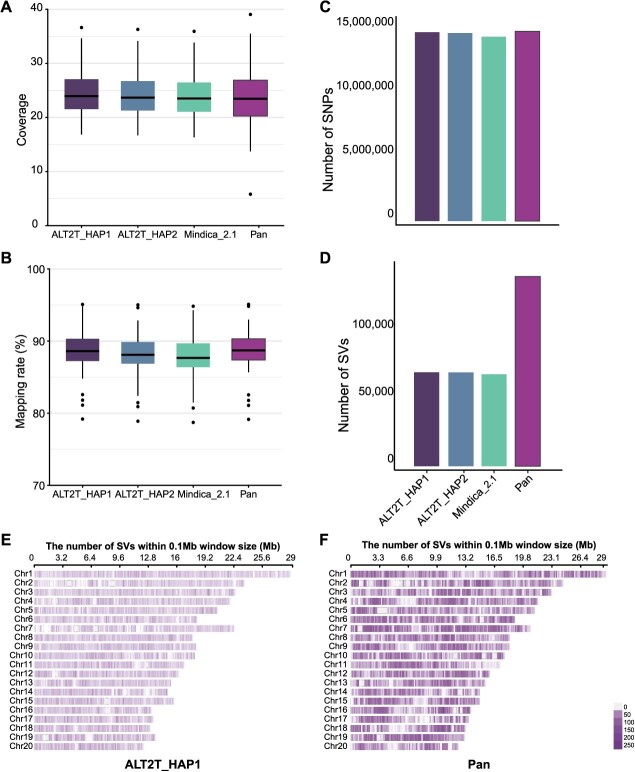
Genomic lanscapes of variations called using different mango reference genomes. (A) Boxplot showing the coverage depth distribution of ALT2T_HAP1, ALT2T_HAP2, Mindica_2.1, and the pangenome (Pan) across 56 WGS samples. The line inside each box denotes median values while the dots at the end of the whiskers represent outliers. (B) Boxplot depicting the mapping rate (%) of different reference genomes across 56 WGS samples. The line inside each box denotes median values while the dots at the end of the whiskers represent outliers. (C) Total number of SNPs detected across 56 WGS samples. X and Y-axis denote the name of the reference genome and SNP number, respectively. (D) Total number of SVs detected across 56 WGS samples. *X* and *Y*-axes denote the name of the reference genome and SV number, respectively. (E) Density map displaying the number of structural SVs detected in ALT2T_HAP1 across each chromosome, within 0.1 Mb windows. Darker shades indicate regions with higher numbers of SVs. (F) Density map displaying the distribution of SVs detected using the pangenome (Pan) across each chromosome, within 0.1 Mb windows. Darker shades indicate regions with higher numbers of SVs

To compare the differences in the accuracy and depth of population analysis in genetic studies using different reference genomes, we utilized four reference genomes: pangenome, ALT2T_HAP1, ALT2T_HAP2, and Mindica_2.1. Whole genome sequencing (WGS) data of 56 mango accessions were mapped to different reference genomes. Among single-reference genomes, ALT2T_HAP1 achieved the highest coverage and depth. However, the pangenome exhibited the best overall performance in mapping rate. At the same time, the lowest coverage depth and mapping rate were calculated for the Mindica_2.1 reference genome (Fig. 2A and B). Similarly, numbers of the most and the least single nucleotide polymorphism (SNPs) were detected using Pangenome and Mindica_2.1 as reference genomes, respectively. However, there was a slight difference (0.47%) in SNPs numbers among ALT2T_HAP1, ALT2T_HAP2, and the pangenome (Fig. 2C; Table S3). However, a significant difference was noticed in SV detection, and almost twice as many SVs were identified using pangenomes than other genomes (Fig. 2D). Among SV types, insertions displayed the highest divergence, being markedly more abundant in the pangenome-based analysis than in linear genome references.

These results highlight the potential of the pangenome to capture more diversity. To further elaborate the results of SVs called using ALT2T_HAP1 and pangenome as a reference are displayed on each chromosome in the form of density maps within 0.1 Mb windows. In the case of ALT2T_HAP1, the highest number of SVs detected in a specific region of the chromosome was 45 (Fig. 2E) while in the pangenome, up to 250 SVs were found in certain regions (Fig. 2F). These results highlight the enhanced power of the pangenome in detecting SVs across the genome.

### Genetic diversity analysis using different reference genomes

For phylogeny analysis of mango cultivars and wild species,four species of the Anacardiaceae family were used as an outer group. The outer group, wild, and cultivars were divided into distinct groups. All wild species except *M. odorata* and *Mangifera wild* specie (Found in Malaysia and regarded as wild on a phenotypic basis, scientific name not confirmed (MWS)) formed a monophyletic group, suggesting a closer evolutionary relationship among these species. At the same time, *M. odorata* and MWS may have undergone distinct evolutionary events or originated from different lineages. However, a slight deviation was observed: *M. siamensis* showed a different lineage in the phylogenetic tree constructed using SNPs called from the pangenome as a reference. Based on phylogenetic trees inferred from SNPs, all the cultivars were further divided into two clades—Southeast Asian origin and Indian origin (Fig. S6A–D). However, differences were noticed in grouping coherence and inter-clade relationships differ between linear genomes and pangenome phylogenetic trees. Pangenome showed stronger separation among groups, suggesting that the pangenome captures more diversity and can further divide clades or monophyletic groups. As well as the choice of reference genome can influence the phylogenetic results. As anticipated, the principal component analysis (PCA) results also divided mango cultivars into two groups, suggesting two centers of mango domestication (Fig. S6A–D). Wild accessions were more clearly divided into two groups. One group was closer to cultivars than others, suggesting a close evolutionary history. According to admixture analysis, two wild species (*M. odorata* and MWS) exhibited admixture (*K* = 4) with Southeast Asian and Indian cultivars. These results coincide with the findings that *M. odorata* is a hybrid between *M. indica* and *M. foetida* [26]. The USA varieties were closely related to Indian cultivars and some leading commercial varieties showed an allelic mixture of Indian and Southeast Asian cultivars (Fig. S6A–D). In general, the choice of reference genome affected the results of phylogenetic relationships but not the results of population differentiation.

Moreover, the SV-based phylogenetic trees (Fig. S7A–D) revealed that *M. odorata* and MWS intermixed with cultivated varieties. Two *M. odorata* accessions were analyzed—one obtained from public data (PRJNA487154, accession SRR11078078) and another sequenced in this study. Notably, only SRR11078078 clustered closely with cultivars, suggesting accession-specific genetic divergence. The intermixing of MWS, a phenotypically wild but taxonomically unconfirmed group from Malaysia, with cultivated mangoes could reflect either historical gene flow or misidentification due to unresolved species boundaries. Differences between SV- and SNP-based phylogenetic trees highlight how distinct variant types capture different evolutionary signals. Earlier, such discrepancies were reported in *Mangifera* classification based on phylogeny. Recently, Ma et al. [27] reclassified *M. hiemalis* as *M. indica*, but in both our SNP- and SV-based phylogenies, it consistently clusters with wild species. This observation aligns with previous studies [21], supporting its placement outside the cultivated clade. These findings highlight the value of integrating multiple variant types to refine *Mangifera* taxonomy.

### Genetic differentiation between cultivated and wild mango populations using different reference genomes

**Figure 3:**
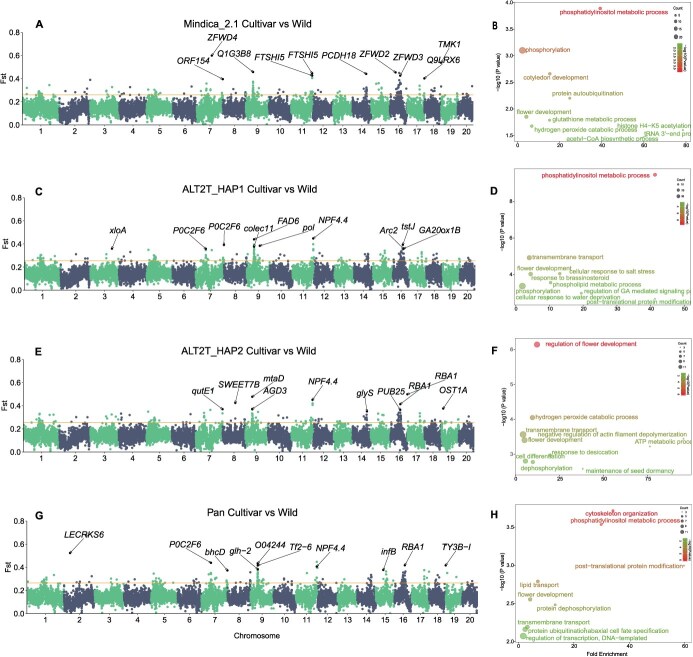
Population differentiation (*F*_ST_) levels *Mangifera* cultivars vs. *Mangifera* wild species. A, C, E, and G denote the Manhattan plot of the *F*_ST_ analysis cultivar vs. wild inferred using SNPs called with Mindica_2.1, ALT2T_HAP, ALT2T_HAP2, and pangenome as a reference, respectively. The x and y-axis denote different chromosome names and *F*_ST_ values in the corresponding windows of each chromosome, respectively. The horizontal yellow line represents a significant threshold level (top 1%). B, D, F, and H denote GO enrichment analysis of genes linked with selective sweep regions detected using Mindica_2.1, ALT2T_HAP, ALT2T_HAP2, and pangenome as a reference, respectively. Different colors of BPs denote the level of enrichment significance

*F*_ST_ (fixation index) is a measure of genetic differentiation between subpopulations. *F*_ST_ analysis revealed considerable variation in the highest *F*_ST_ values and their chromosomal locations across different reference genomes. For instance, Mindica_2.1 displayed the highest *F*_ST_ value of 0.599 on chromosome 7, ALT2T_HAP1 recorded 0.447 on chromosome 11, ALT2T_HAP2 showed 0.543 on chromosome 17, and the pangenome demonstrated the highest value of 0.674 on chromosome 2. To highlight the influence of reference genome on genetic differentiation, the top 10 genetically differentiated regions and linked genes are shown (Fig. 3A, C, E, and  G). The location and length of regions, the number of genes, and the type of genes linked with genetically differentiated regions were different. For instance, the pangenome displayed a higher *F*_ST_ value (0.523) on chromosome 2. However, the other three genomes do not show genetic differentiation in this region. Three genes relate to this region; one representative gene, “*LECRKS6*” is marked (Figs 3G and S8). The L-type lectin receptor kinases (LecRK) genes have been well studied for their roles in fungus (Phytophthora) and bacterial (*Pseudomonas syringae*) stress resistance [28]. Another gene *P10582* in the region codes for DNA/RNA polymerase proteins that are core components of the RNA-directed DNA methylation pathway, which directs epigenetic silencing of transposons and stress-responsive genes through small RNA-mediated DNA methylation [29, 30]. These results indicate that different reference genomes can lead to significant discrepancies in identifying regions of genetic divergence. Moreover, chromosomes 9 and 16 showed similarities across all genomes, with more regions linked to selective sweeps, suggesting they have experienced stronger selection pressures. GO enrichment analysis was performed to justify our results further as this is very sensitive to small variations in gene numbers or gene annotations [31]. Based on GO enrichment analysis, the top five BPs varied among genomes. These results indicate that using different genomes as a reference significantly influences genetic differentiation results (Fig. 3B, D, F, and  H). However, GO terms linked with flower development (GO:0009908), phosphatidylinositol metabolic process (GO:0046488), and transmembrane transport process (GO:0055085) were constantly enriched among genomes. These enriched GO terms suggest functional adaptations in flowering and stress tolerance, which may underlie selection in mango domestication. Phosphoinositides, as lipid signaling molecules derived from phosphatidylinositol, play critical roles in abiotic and biotic stress signaling pathways, including responses to salinity, drought, heat, and pathogen infection [32–35]. Notably, *AtPI4Kγ3* overexpression enhanced salinity and ABA tolerance while delaying flowering, indicating crosstalk between stress signaling and flowering time regulation [36], suggesting crosstalk between stress signaling and flowering time regulation. Membrane transporters, essential for ion and nutrient homeostasis, further contribute to these adaptive processes by regulating water balance, nutrient uptake, and cellular responses under environmental stress [37]. This information revealed that enriched processes are interlinked and required for the normal growth and development of plants under changing environments. The consistent enrichment of these processes indicates that regions linked with these processes are under strong selective pressure in wild and cultivated populations of mango. Further suggests that linked traits like flowering, stress tolerance, and adaptability to changing environmental conditions are critical for fruit production and yield.

### Artificial selection in mango domestication using different reference genomes

**Figure 4:**
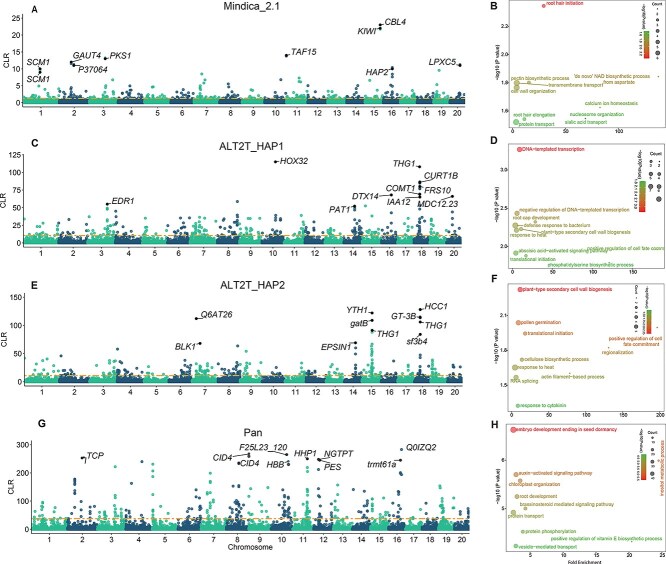
Artificial selection during mango domestication detected using SweeD. A, C, E, and G denote Manhattan plot of SweeD analysis of mango cultivar inferred using SNPs called using Mindica_2.1, ALT2T_HAP1, ALT2T_HAP2, and pangenome as a reference genome, respectively. The x and y-axis denote different chromosome names and CLR values, respectively. The horizontal yellow dashed line represents a significant threshold level (top 1%). B, D, F, and H denote GO enrichment analysis of genes linked with selective sweep regions detected using Mindica_2.1, ALT2T_HAP, ALT2T_HAP2, and pangenome as a reference genome, respectively. Different colors of BPs denote the level of enrichment significance

Little research has been performed to identify the positive selection in mango cultivars during selective breeding. Selective sweeps can uncover loci under positive selection linked with important adaptive traits. To gain deeper insights, we identified selective sweeps using different reference genomes (Mindica_2.1, ALT2T_HAP1, ALT2T_HAP2, and pangenome) and conducted GO analysis to infer their roles. We focused on the top 1% of selective sweep regions to investigate potential selective signals. All genomes showed different trends of composite likelihood ratio (CLR) values. For example, the maximum range of CLR values was 25, 120, 150, and 300 for Mindica_2.1, ALT2T_HAP1, ALT2T_HAP2, and pangenome, respectively. The genome-wide distribution of selective sweep regions varied across genomes, with each suggesting different regions under selection. Mindica_2.1 and pangenome have identified fewer and more regions under selection, respectively (Fig. 4 A, C, E, and G). Interestingly, despite being haplotypes with similar genome quality, ALT2T_HAP1 and ALT2T_HAP2 also showed discrepancies in identifying regions under selection. ALT2T_HAP2 identified two selective regions on chromosome 7, while ALT2T_HAP1, used as a reference genome, did not reveal any selective regions in the same area. Similarly, chromosome 15 presented multiple selective sweep regions and numerous linked genes in ALT2T_HAP2. The divergent selective regions in ALT2T_HAP1/HAP2 are due to allele-specific gene content (4008 vs. 467 unique genes) and SV distribution. The 202 additional translocations in HAP1 could reorganize regulatory landscapes. However, an almost identical distribution pattern was observed between ALT2T_HAP1 and ALT2T_HAP2 on chromosome 18 (Fig. 4C and E). The Mindica_2.1 genome exhibited more selective sweeps on chromosome 16, whereas the pangenome identified more sweeps on chromosomes 10, 16, and 19 (Fig. 4A and G).

**Figure 5:**
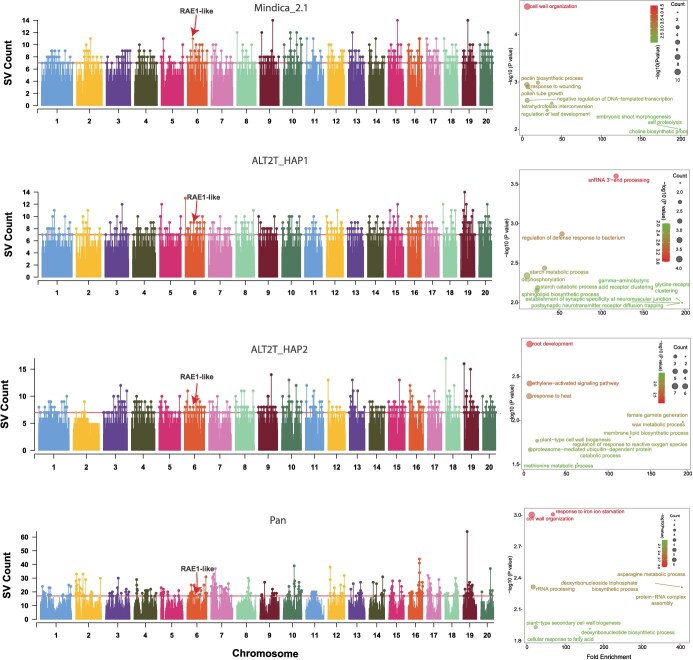
SV hotspots based on four different mango reference genomes. A, C, E, and G represent Manhattan plots showing the distribution of SV hotspots across chromosomes called using Mindica_2.1, ALT2T_HAP1, ALT2T_HAP2, and Pangenome as a reference genome, respectively. SV hotspots are defined as regions within the top 1% of SV counts. The horizontal red line represents a significant threshold level (top 1%). B, D, F, and H denote GO enrichment analysis of genes linked with SV hotspots detected using Mindica_2.1, ALT2T_HAP1, ALT2T_HAP2, and Pangenome as reference genome, respectively. Different colors of BPs denote the level of enrichment significance. A conserved SV hotspot linked with *RAE1*-like gene (Q38942), associated with fruit acidity, is marked on chromosome 6 across all references

For comparative purposes, the top ten regions and their associated genes across all genomes are highlighted. To further support the results, GO enrichment analysis was conducted for genes linked to selective sweep regions. GO enrichment analysis also suggested notable diversity in the top enriched BPs across the different genomic contexts (Fig. 4B, D, F, and  H). For example, the Mindica_2.1 genome (Fig. 4B) showed unique enrichment in root hair initiation and pectin biosynthesis process, suggesting adaptations for enhanced nutrient acquisition and abiotic stress responses. ALT2T_HAP1 (Fig. 4D) was specifically enriched for DNA-templated transcription (GO:0006351), pointing to haplotype-specific regulatory functions. While ALT2T_HAP2 (Fig. 4F) identified processes such as plant−type secondary cell wall biogenesis, pollen germination, and cytokinin response, pointing to haplotype-specific regulatory functions. Pangenome (Fig. 4G) highlighted embryo development and auxin-activated signaling pathway. The diversity in BPs suggests that each genome highlights different aspects of functional genetic diversity with potential phenotypic implications. Despite the observed differences, the processes linked with root development and functionality (GO:0048364), plant-type secondary cell wall biogenesis (GO:0009834), and response to heat (GO:0009408) were consistently enriched across the genomes. The consistency of these processes (putative selective sweeps) indicates that selection pressure has favored populations with optimized growth and stress adaptation mechanisms. We surmise that these processes might have a central role in the evolutionary dynamics of the mango.

### SV hotspot detection across populations using different reference genomes

We further investigated the influence of reference genomes on the BPs linked with SV regions. Using the sliding window method [38], the windows with the top 1% SV index were designated as SV hotspots. The regions above the red horizontal line in Manhattan plots show SV hotspots. The pangenome reference genome revealed a greater number of SVs in specific regions of SV hotspots compared to the other reference genomes. In the pangenome, one single region contained 17 to 64 SVs, whereas for the other genomes, the maximum number of SVs identified in a single region ranged from 7 to 17. This suggests a significant difference in SV density within SV hotspot regions (Fig. 5A, C, E, and  G). Furthermore, genes overlapping with SV hotspots regions were subjected to GO analysis. The BPs linked to SV hotspot regions varied across genomes. For example, when using Mindica_2.1 as the reference genome, the linked genes were enriched in functional terms related to cell wall organization, pectin biosynthesis process, and response to external stimulus (biotic and abiotic) (Fig. 5B). For ALT2T_HAP1, the BPs including snRNA 3′ end processing, defense response to the bacterium, starch catabolism and metabolism, and dephosphorylation were enriched (Fig. 5D). ALT2T_HAP2 SV hotspots regions linked genes were enriched in root development, ethylene-activated signaling pathways, and heat response (Fig. 5F).

Likewise, pangenome SV hotspot regions were prominently enriched in cell wall organization and RNA processing BPs (Fig. 5H). Despite discrepancies, several SV hotspots and their associated genes were consistently identified across all reference genomes. Notably, on chromosome 6, overlapping SV hotspot regions were observed in all genomes, flanking the gene *Alphonso06G000815* (Mindica_2.1) and its homologs in ALT2T_HAP1 and ALT2T_HAP2. These genes correspond to the UniProt ID Q38942, encoding an RAE1-like protein, which has been functionally characterized in citrus species and plays a well-documented role in regulating fruit acidity levels [39]. Protein BLAST analysis confirmed high similarity between the mango and citrus proteins (98%), reinforcing their potential functional equivalence. The consistent enrichment of SVs in this genomic region across multiple references, combined with its functional annotation, suggests that SV near RAE1-like genes may contribute to regulatory diversity influencing fruit acidity or sweetness. This highlights a promising target region for future functional validation. Moreover, the BPs related to root, cell wall, and response to external stimuli were commonly enriched across the genomes. This indicates that SV-hotspot regions are linked to critical physiological functions in plants, possibly playing a potential role in the adaptation and regulation of essential biological functions. Moreover, processes linked with plant cell wall functioning and stress responses were consistently enriched in regions of genetic differentiation, selective sweeps, and SV hotspots. The constant enrichment of these processes suggests that linked regions are under strong selective pressure, possibly due to their potential roles in normal growth and development and stress tolerance.

## Discussion

The selection of reference genomes plays a critical role in population genomic analyses, and poorly assembled or fragmented genomes can introduce reference biases [3, 40, 41]. Our study provides the first high-quality haplotype-resolved T2T genome assembly of mango, and issues of previous assemblies, such as gaps and unidentified centromeres have been resolved. Our population genomic analyses using different reference genomes suggest that pangenomes can reduce reference bias in upstream and downstream analysis. First, pangenome indicated high mapping and coverage rates for variant calling with slight changes (0.47%) in SNP numbers and significantly higher SV (~50%) numbers. Mostly, SNP detection displays only small variations, while SV detection is influenced dramatically by the choice of reference genome [42–44].

Second, in downstream analysis, the PCA results based on different reference genomes showed similar results, but phylogenetic and admixture analysis displayed slight changes. Phylogenetic studies are sensitive to the choice of the reference genome, as even minor changes can alter tree branches or clades [45]. Changes in accession distribution were also observed in phylogenetic trees based on SNPs and SVs. Furthermore, genetic differentiation and artificial selection analyses in mango domestication identified more regions under selection when using the pangenome. Third, GO analysis of SV hotspots and regions under selection highlighted distinct biologically enriched processes for each genome, implying that the choice of reference strongly influences functional inferences. Our study suggests that reference bias dramatically impacts genomic analyses, and pangenome is a better choice than high-quality linear genomes.

### Reference bias has dramatic impacts on genomic analyses

Reference bias begins at the mapping stage of WGS, where reads are aligned to the reference genome, potentially leading to inaccuracies in detecting genetic variation, which is more prevalent in SV detection [42]. Detection of a significantly higher number of SVs using pangenome can have two possible explanations: first, the pangenome captures greater genetic diversity, leading to the identification of more SVs. Second, there are inherent differences in the methods used for SV detection between linear genomes and pangenomes. This highlights the importance of using consistent methods when performing population-level SV detection across both genome types. Accurate estimation of SVs is more important, as individual SVs can influence more gene functions than SNPs [13, 42] due to larger genomic SVs. These results endorse the need for specialized algorithms to detect larger SVs, as many of these variants may not be represented in linear reference genomes, leading to an underestimation of population variation [42].

Minor changes in phylogenetic results and similar PCA analysis results for different reference genomes endorse that phylogenetic studies are more responsive to reference bias, even minor changes can alter tree branches or clades [45]. While PCA is less responsive to reference bias as it considers general trends of variation rather than specific alignments. These results indicate that the pangenome can reveal hidden variations ignored by single reference genomes. Pangenome highlighted the allelic mixture of two wild species (*M. odorata* and *M. siamenis*) with Chinese cultivars, suggesting that pangenome can reveal more insights into genetic diversity. The discrepancies in identifying selective sweep regions and linked genes across genomes emphasize that single linear references may miss critical variation, particularly from less common haplotypes. For example, the *LECRKS6* gene linked to stress resistance was detected in the pangenome but not in the linear references, suggesting the limitations of single genomes in harnessing full diversity. Such differences can ignore key adaptive traits under selection. These differences arise from genome assembly quality (e.g. Mindica_2.1 vs. T2T assemblies), extensive allele-specific gene content between ALT2T_HAP1 and HAP2 (4008 vs. 467 unique genes), and the enhanced ability of the pangenome to capture broader genetic diversity. These findings align with previous reports in apple, grape, and pear [38, 44, 46]. Conversely, similarities in detecting regions under selection on chromosomes 9 and 16 indicate that these chromosomes underwent stronger selection pressures during domestication, regardless of the reference used. Similarly, the higher CLR values in the pangenome suggest that it captures a broader range of adaptive traits, supporting previous findings that indicate pangenomes provide better resolution for identifying evolutionary signals [47].

Differences in top-enriched BPs associated with regions under selection across different reference genomes endorse previous findings that GO analyses are more responsive to the number and kind of genes [31]. However, despite discrepancies, BPs linked with flower development, root functioning, phosphatidylinositol metabolic process, transmembrane transport process, and response to heat were identified as putative selective sweeps in genomic scans. The constant enrichment of flower development (GO:0009908), phosphatidylinositol metabolic process (GO:0046488), and transmembrane transport (GO:0055085) suggested their critical roles in the domestication and adaptation of mango. As anticipated, top biologically enriched processes linked with SV-hotspot region were also different for each genome (Fig. 5B, D, F, and  H). These findings imply that the choice of reference strongly influences these functional results.

### Pangenome as the new golden standard as a reference genome in genomic studies

Better performance of pangenome in variant calling, downstream analyses, and functional inferences, endorse its utilization as the new golden standard for reference genome in genomic studies. In selecting a suitable reference genome, two key factors must be considered: the quality of the genome and its genetic relevance to the population under study. Despite these advancements, comprehensive bioinformatics analyses are required to perform detailed variant calling using large samples from diverse populations. Our work reviewed the dramatic impacts of mango pangenome on read mapping, variant calling, and population genomics analysis. We highlighted its novel potential in reducing reference bias and advocate for using the pangenome as a reference in genomic studies.

## Material and methods

### Sample collection

Healthy and fresh leaf samples were collected from mango cultivar “Alphonso” being grown at National germplasm resource repository of mango, Tiandong, China. After collection samples were immediately immersed in liquid nitrogen. The samples were stored at −80°C and subsequently sent to the company for ONT ultra-long, HiFi, and Hi-C sequencing.

### Library preparation and DNA sequencing

The Plant Mini Kit (Qiagen) was used to extract high-molecular-weight genomic DNA, following the manufacturer's instructions. The library was prepared with the Oxford Nanopore SQK-LSK109 kit and sequencing was performed on the PromethION ONT platform for getting ONT ultra-long sequences. PacBio HiFi sequences were obtained using single-molecule real-time sequencing on the PacBio Sequel II platform, employing circular consensus sequencing (CCS) with default settings. For Hi-C, the library was generated as previously described [48] and the Illumina HiSeq X Ten platform was used for sequencing.

### Genome assembly generation and quality assessment

HiFi and Hi-C reads were *de novo* assembled using Hifiasm (v0.16) [49]. The initial output of Hifiasm generated a pair of components “p_ctg” containing the phased haplotypes. Afterward, pseudochromosomes were structured, confirmed, grouped, and anchored with Hi-C reads using Juicer (v1.5) and 3D-DNA (v180922) [50, 51], with Hi-C read alignment performed using Juicer’s built-in aligner. Juicebox (v1.11.08) and Python script (getgaps.py) were used for the manual adjustment of the interactive heatmap [52] and for detecting gaps in pseudochromosomes, respectively. Furthermore, NextDenovo (v2.4.0) was used to assemble ONT reads. The assembled contigs and ONT reads were manually adjusted and mapped to the final haplotypes using Minimap2 (v2.24) [53]. In this way, gap-free haplotype resolved assembly of all 20 chromosomes was obtained, and both haplotype assemblies' accuracy was confirmed using the KAT program [54, 55]. *k*-mer spectra analysis was performed to assess completeness and identify potential assembly artifacts. *K*-mers present in the reads but missing from the assembly indicated possible sequence loss, while *k*-mers overrepresented in the assembly suggested false duplications. This allowed us to evaluate both completeness and assembly error rates.

The heterozygosity level and genome size were estimated using *k*-mer distribution approach with jellyfish and GenomeScope2.0, using a *k*-mer size of 21 [56, 57]. Furthermore, BUSCO (v5.2.2) with the Embryophyta_odb10 database was used for checking completeness of assembles [58]. The continuity and integrity of genome assemblies were estimated using the N50 metric. Moreover, a process called "calc_switchErr" (https://github.com/tangerzhang/calc_switchErr) was used to identify switch and hamming errors in genome assembly. This workflow relied on a "true" phased SNP dataset produced using HiFi and Hi-C reads.

### Annotation of genes and TEs

The TEs were detected using the Extensive De-Novo TE Annotator (EDTA, v2.0.0) with its default settings [59]. Genome annotation was performed using Genome-Wide-Annotation-Pipeline (https://github.com/unavailable-2374/Genome-Wide-Annotation-Pipeline). InterProScan (v5.29) was used to identify potential protein domains for functional annotation [46].

### Telomere and centromere identification

Telomere sequences were identified using the TeloExplorer option of quart quarTeT (https://github.com/aaranyue/quarTeT) Toolkit with default parameters. It requires genome only in FASTA format as an input. TeloExplorer integrates the “explore” and “search” tools from the telomere identification toolkit (tidk) (https://github.com/tolkit/telomeric-identifier), automating the process and providing more accurate results. Centromeres were identified using the bioinformatic tool CentIER (https://github.com/simon19891216/CentIER) with default settings [60]. Due to the high variability and evolving characters, centromere prediction is still challenging [61]. Different types of TEs were extracted from the TEanno.gff3 file using the “grep” command in the Linux system to further validate the predictions. The extracted TE files were visualized alongside the genome annotation file in IGV (v2.12.3). Gypsy and LINE TEs were consistently enriched in regions with low gene density across all chromosomes. Regions with a high abundance of both TEs and low gene content were predicted as centromere boundaries.

### Comparative genomic analysis

Whole-genome alignment was performed among the ALT2T_HAP1, ALT2T_HAP2, and Mindica_2.1 using MUMmer (v4.0.0) with parameters “NUCmer -c 90 -l 40” [62]. The alignment blocks were then filtered using “deltafilter” in the one-to-one alignment mode. Detailed comparison results were extracted using the parameters “show-coords -T -q -H”. After that, the alignment blocks were filtered in the one-to-one alignment mode using "delta filter." The parameters “show-coords -T -q -H” were used to retrieve comparative analysis results. Furthermore, structural rearrangements and collinear orthologues were identified using Synteny and Rearrangement Identifier (SyRI) (https://github.com/schneebergerlab/syri) [63]. Next, SVs were identified through filtration. Initially, SNPs were extracted from the SyRI VCF output file using bcftools to get a SNPs file. Subsequently, “grep” function was used for getting different types of SVs i.e. for deletions (CPL/DEL), inversion-related events (INV/INVTR/INVTRAL), translocations (TRANS/TRANSAL), and insertions (INS/CPG) in the VCF file. The graphical display Plotsr [64] was used.

### Pangenome construction and analysis

The pangenome was constructed with a Minigraph-Cactus pipeline [65] by using five high-quality genomes. Mindica_2.1 was used as a reference in the pangenome. For SNP calling, Vg giraffe (v1.49.0) [66] was used with default parameters to map WGS sequences against the pangenome. The resulting bam files were rehearsed following a series of commands and manually adding the line "@RG ID." Finally, SNPs were identified using GTX (v2.2.1), following the same parameters as linear genomes. Moreover, population-level SVs were identified with PanGenie (https://github.com/eblerjana/pangenie) using the resulting MC.vcf file.

### SNP and SV calling and filtration

The genome sequencing (WGS) data of 51 mango accessions, including 47 cultivars and 4 wild types, was downloaded from the NCBI Sequence Read Archive (PRJNA487154) for SNP calling. Additionally, WGS data for four members of the Anacardiaceae family including *Bouea macrophylla*, *Spondias dulcis*, *Toxicodendron pubescens*, and *Pistacia vera* (DRR186129, DRR186130, ERR7620143, SRR4453368) was downloaded for use as an outgroup. In addition to these, the WGS of five wild mango species was generated as part of this study (Supplementary Table S4). SNPs were identified against four different genomes (Mindica_2.1, ALT2T_HAP1, ALT2T_HAP2, and pangenome) to investigate the effect of the reference genome in SNP calling and subsequent analyses. The BAM files were generated using GTX's mapping function with reference genome indexing by BWA. SNPs were identified using the GTX "vc" function. Filtering was applied with parameters such as—max-missing 0.9—minQ 30 —minGQ 30—min-alleles 2—max-alleles 2—minDP 5—maxDP 100—remove-indels—recode—recode-INFO-all [67].

SV detection at the population level was conducted using DELLY v1.1.6 [68] with Mindica_2.1, ALT2T_HAP1, and ALT2T_HAP2 as reference genomes. First, the "call" and "merge" functions were used to generate .bcf files. Then, the "call" function was run again with the "-v" parameter on the merged .bcf files. Afterward, BCFTools v1.13 was used to merge the .bcf files. The SVs were filtered using the "filter" function in DELLY, and low-quality SVs were removed according to criteria outlined in a previous study [69].

### Population structure analysis

To investigate the relationships among mango species principal component, phylogenetic, and population admixture analyses were performed. PCA was performed using PLINK (v1.90b6.21) [70]. For the SNP-based phylogenetic tree, input sequences were generated using a custom Python script (vcf2other.py). Then FastTree with the GTR + G model and nucleotide sequences was used for tree generation between *Mangifera* accessions and Anacardiaceae members (outer group). Admixture analysis was performed using “admixture v1.3.0” with k from 2 to 10, and R package was used to display the results [71, 72]. SV-based phylogenetic trees were constructed using PhyloForge with default parameters, utilizing SV calls in VCF format as input [73]. To infer phylogenetic relationships among mango genomes (Fig. S9), a tree was constructed using *P. vera* (assembly GWHBKLC00000000 from NGDC) as the outgroup. Single-copy orthologous genes shared among five mango genomes and the outgroup were identified using OrthoFinder v2.5.4 [74] based on their longest protein-coding sequences. These orthologues were aligned with MAFFT v7.310 [75], and the resulting alignments were concatenated. The final phylogenetic tree was generated using PhyML v3.3.20190909 [76] with the JTT model and the following parameters: -f e -v 0.576 -a 0.886—nclasses 4—search SPR -t e. All trees were visualized using the online tool iTOL [77].

### Genome scanning for selective sweep signals

SweeD software (v3.3.1) was used based on the CLR test to identify signatures of domestication in mango cultivars [78]. We also calculated the population divergence statistic (*F*_ST_) to investigate the selection signals across the whole genome. A 20-kb sliding window with a 20-kb step was applied to quantify *F*_ST_ using VCFtools software (v0.1.13) [67]. Sliding windows with the top 1% values were picked as candidate selective signals.

### SV hotspot identification across populations

SVs detected across mango populations using different genomes as a reference were analyzed and a sliding-window-based approach was used to detect SV hotspots. A 10-kb sliding window with a step size of 10 kb was created to move across each chromosome. The SV index for each sliding window was calculated by dividing the number of SVs in the window by the total number of SVs in all samples within the window. After calculating the SV index for each window, the windows were ranked based on their SV index values, and the top 1% of windows were designated as SV hotspots.


\begin{equation*} SVIi=\sum_{j=1}^{ni}Cj \end{equation*}

where SV*Ii* = the index for each sliding window, *ni* = The number of SVs in the *i*th window, and *Cj* = total count of the *j*th SV in all samples within the *i*th window [38].

### GO enrichment analysis

The uniprot_sprot.fasta file was downloaded from the UniProt database (https://www.uniprot.org/help/downloads), and selected protein sequences were compared against it using the BLASTP option. BLAST results were filtered using the criteria of E-value <1e-10, identity >70%, and coverage >70%. Gene IDs from the BLAST results were submitted to the DAVID platform (https://david.ncifcrf.gov/tools.jsp) for GO analysis. The top ten BP pathways were visualized using the ggplot2 R package. Furthermore, the same gene ID list was submitted to UniProt’s ID mapping tool (https://www.uniprot.org/id-mapping), and the Excel “vlookup” function was used to map the functional annotation of the candidate genes [69].

## Supplementary Material

Web_Material_uhaf166

## Data Availability

Data have been deposited in NCBI and NGDC under the following bioproject numbers: PRJNA1218505, PRJNA1218506, PRJNA1218522, PRJCA035721, and PRJCA035722. Genome assemblies and annotations are also available on the web site (https://zenodo.org) under number 15798809. All other data are included in the article and/or supporting information.
