# Supplementary material for: Mango pangenome reveals dramatic impacts of reference bias on population genomic analyses
Source: Hortic Res. 2025 Jul 1;12(9):uhaf166. doi: 10.1093/hr/uhaf166 (PMC12344551; doi:10.1093/hr/uhaf166)
Supplement: Web_Material_uhaf166 [file web_material_uhaf166.zip › Supplementary Information_Figures.docx]

**Supplementary Information**

**
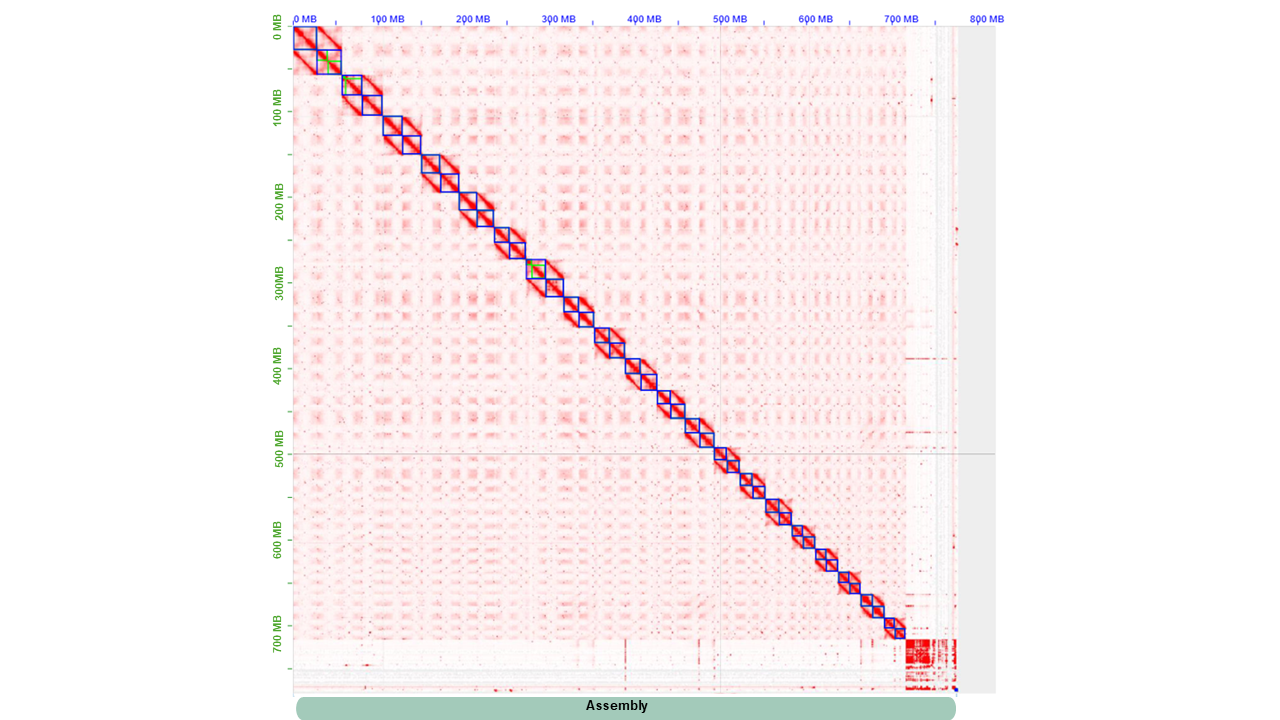
**

**Figure S1.** Hi-C heat map of mango chromosomes interactions. Blue boxes represent individual chromosomes.


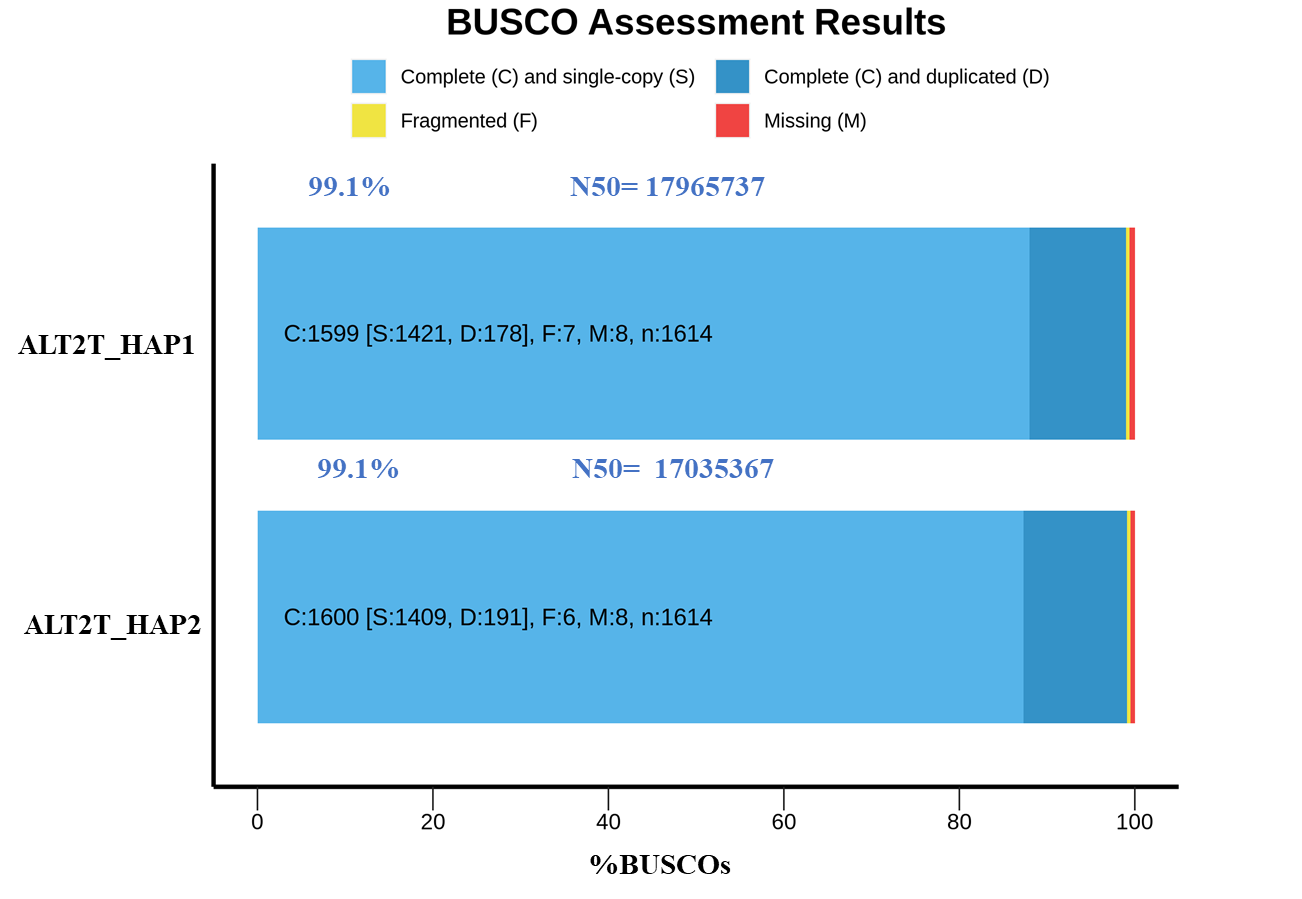


**Figure S2.** BUSCO assessments of ALT2T_HAP1 and ALT2T_HAP2. Embryophyta_odb10 database was used.


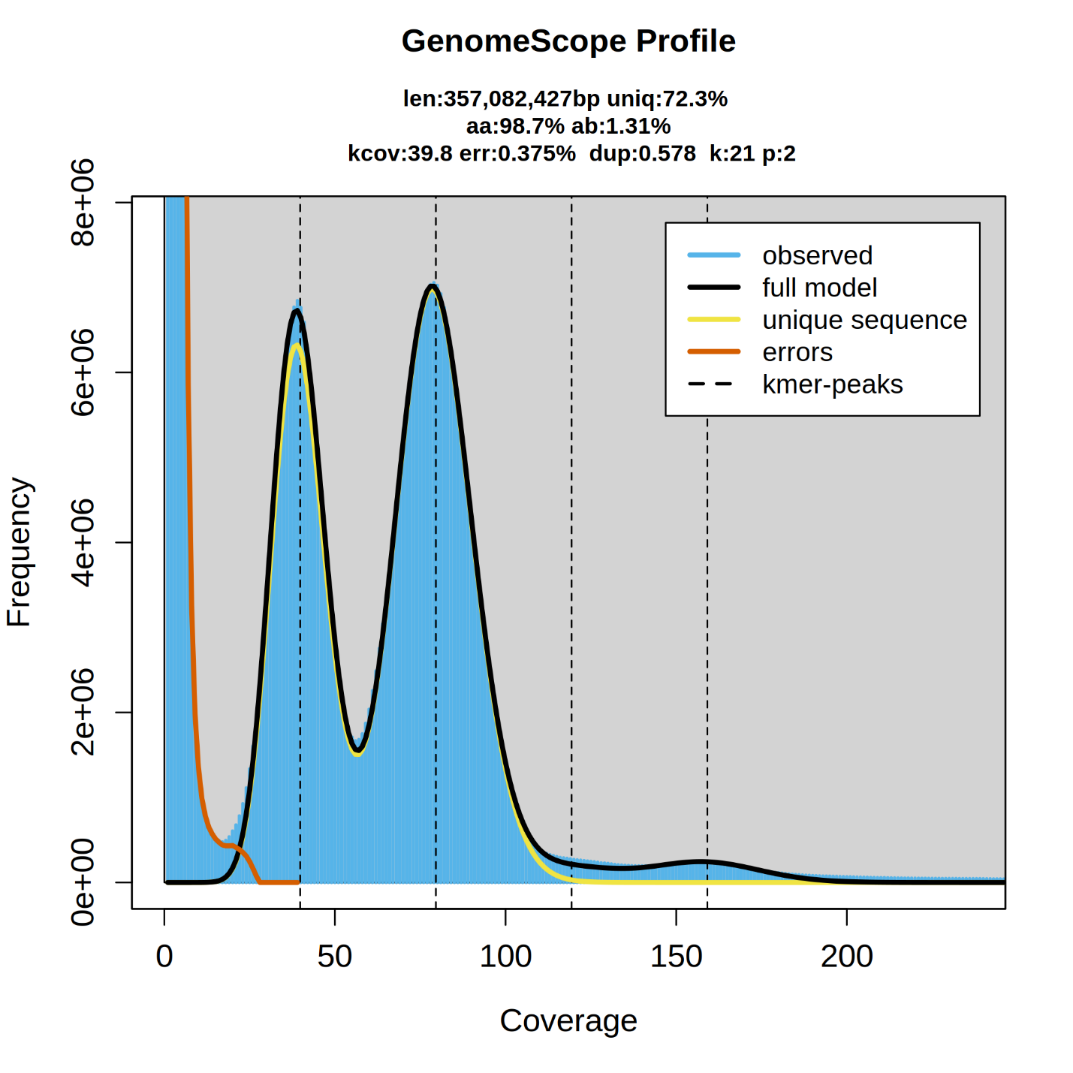


**Figure S3.** K-mer profile (K=21) analysis performed with Genome Scope for genome size and heterozygosity estimation. The relative heights of the peaks are proportional to the heterozygosity of the genome. The heterozygosity was estimated as 1.31%

**A**


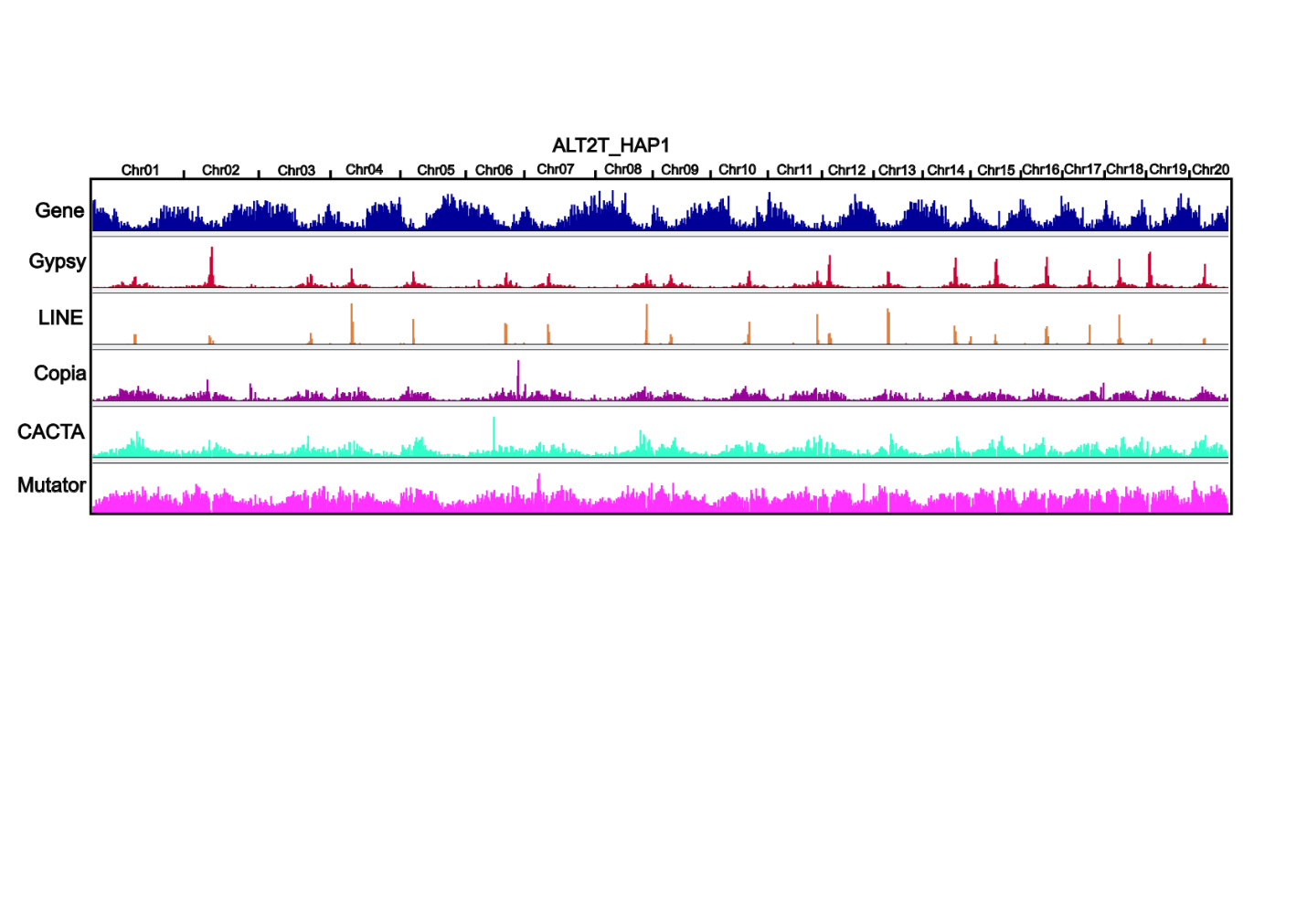


**B**


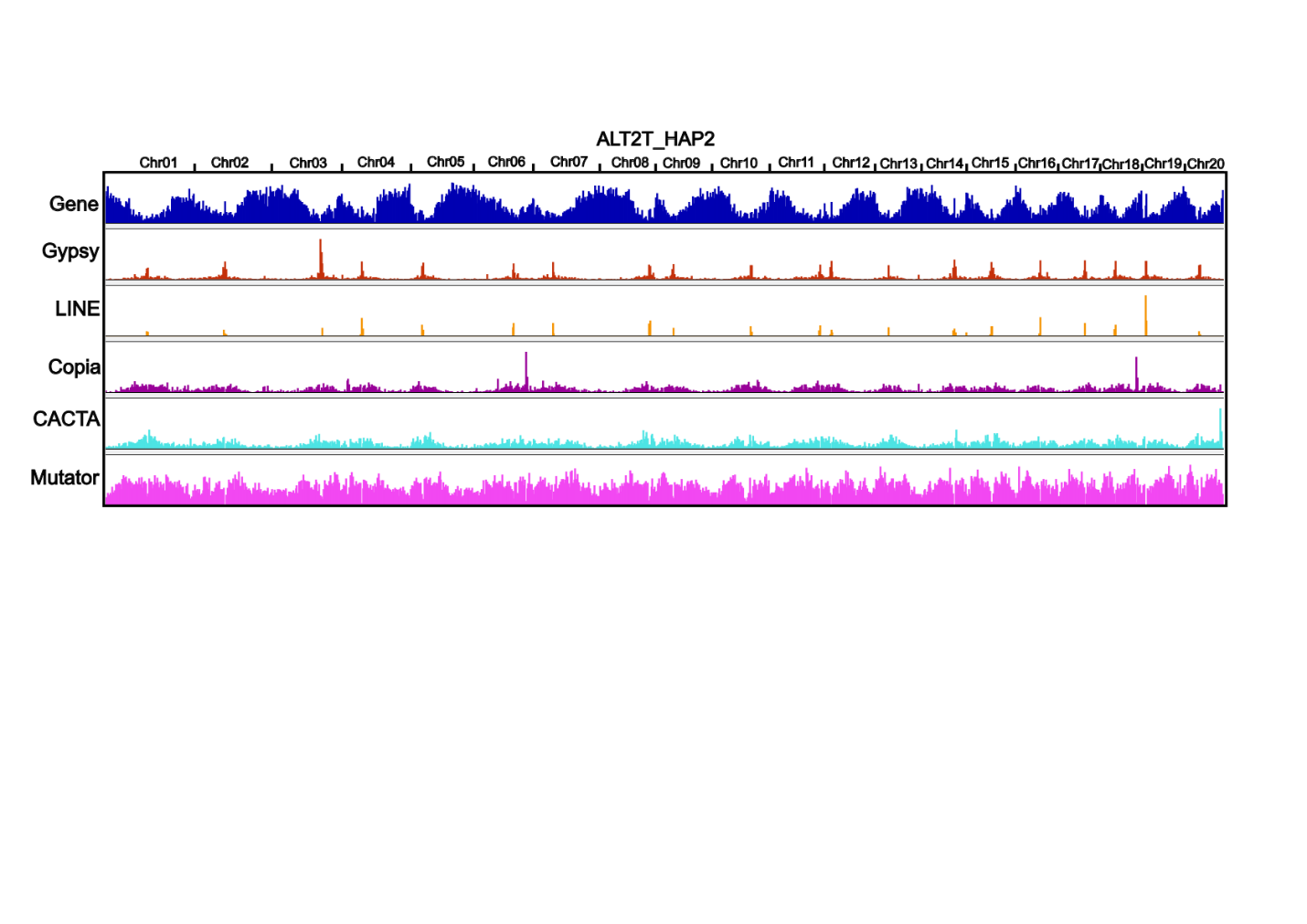


**Figure S4.** Visualizing transposable elements (TEs) throughout the entire genome using IGV. **A.** ALT2T_ HAP1. **B.** ALT2T_HAP2. The horizontal axis denotes the 20 chromosomes, and the vertical axis denotes different types of TEs

**A**


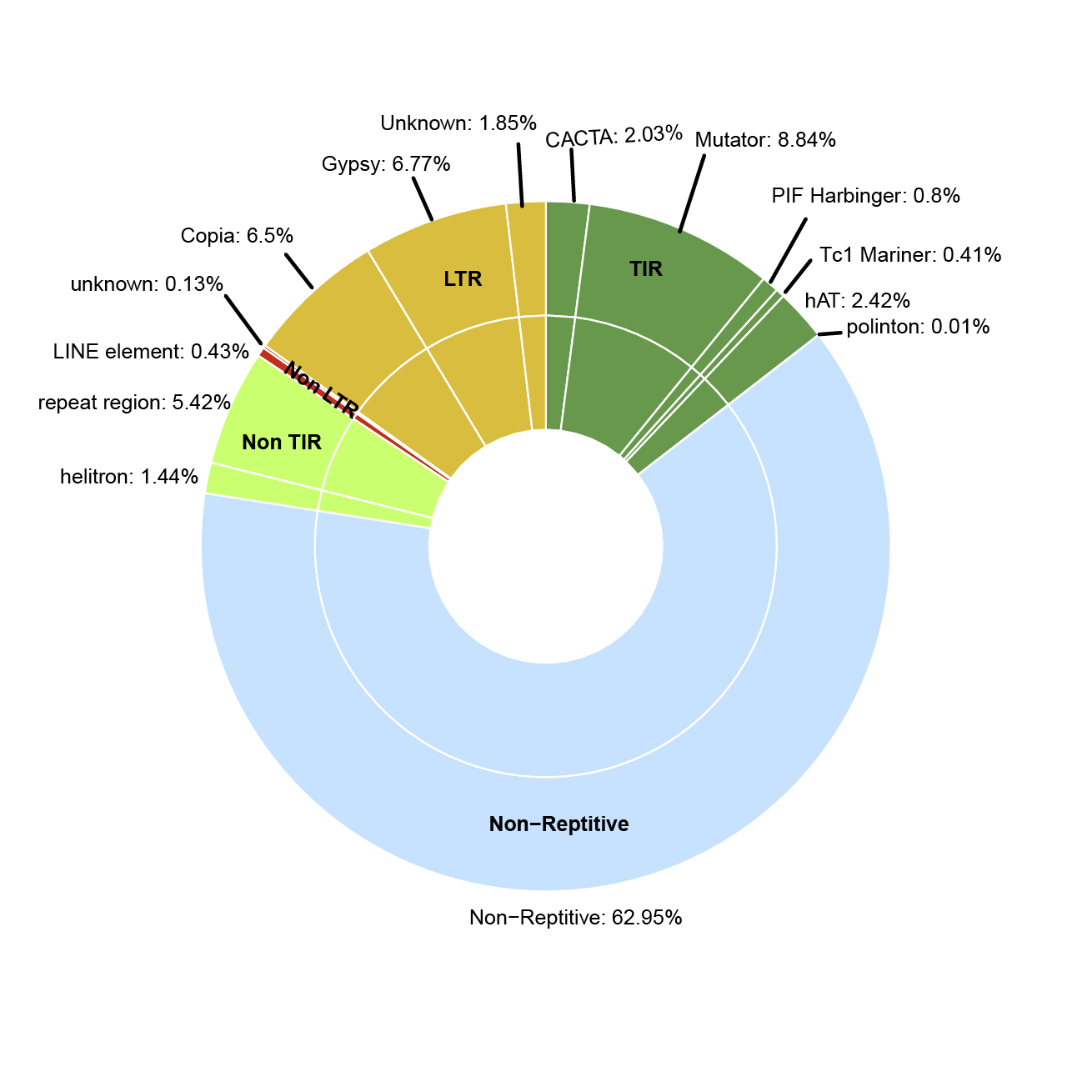


**B**


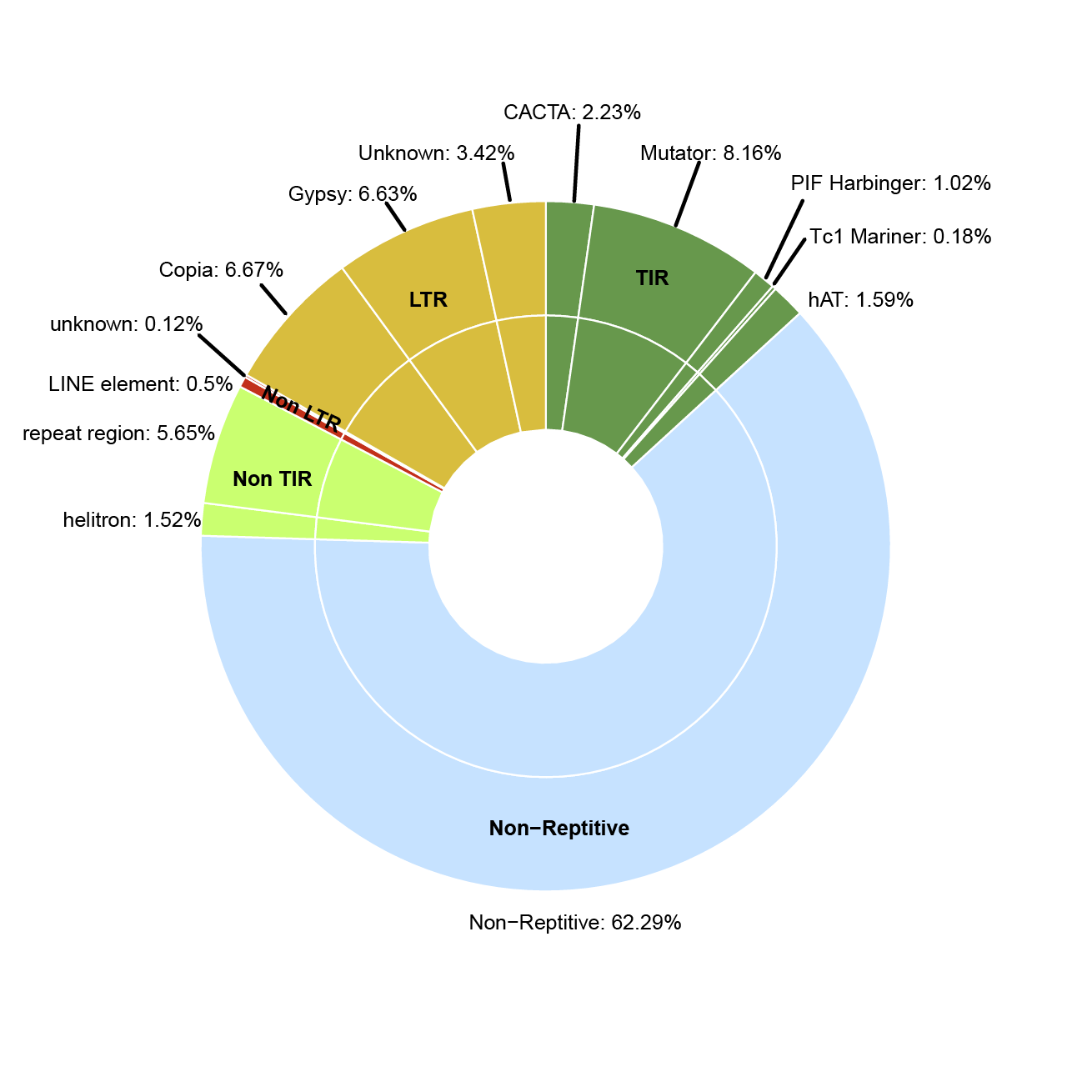


**Figure S5.** Types and percentages of different transposable element families detected in the **(A)** ALT2T_HAP1 and **(B)** ALT2T_HAP2 genomes.


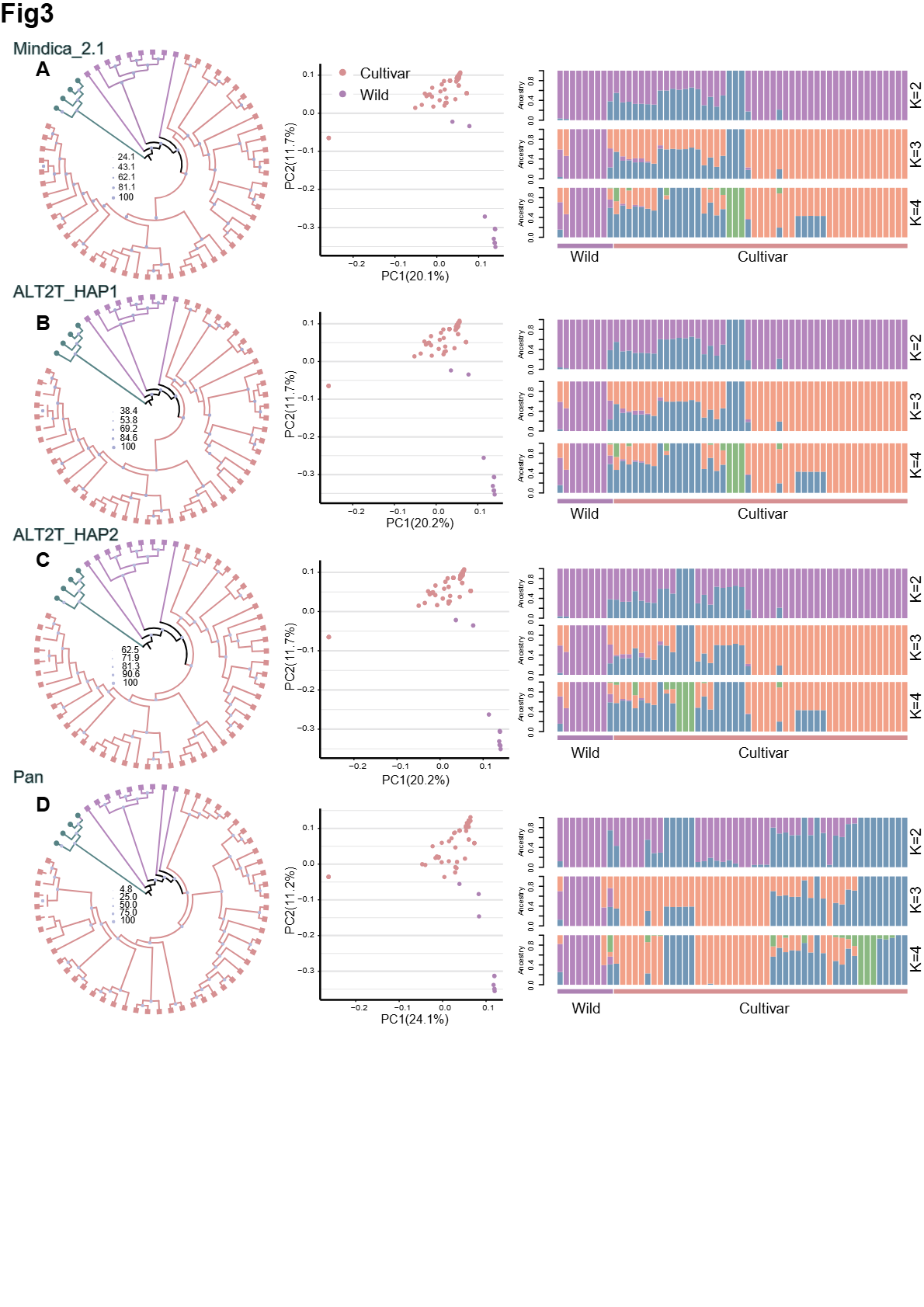


**Figure S6. Genomic diversity analysis using SNPs from various mango accessions called against four different reference genomes**. The left panels display a neighbor-joining phylogenetic tree constructed from SNPs, where cultivars, wild types, and the outgroup are represented in orange, blue, and green, respectively. The middle panels show a Principal Component Analysis (PCA) that highlights the separation between cultivars and wild types along the first two principal components (PC1 and PC2). The right panels present the admixture analysis, where different colors indicate distinct populations, and the length of each color segment in the vertical bars corresponds to the proportion contributed by ancestral populations. K represents the number of clusters. **(A)** Mindica_2.1, **(B)** ALT2T_HAP1, **(C)** ALT2T_HAP2, and **(D)** Pangenome.


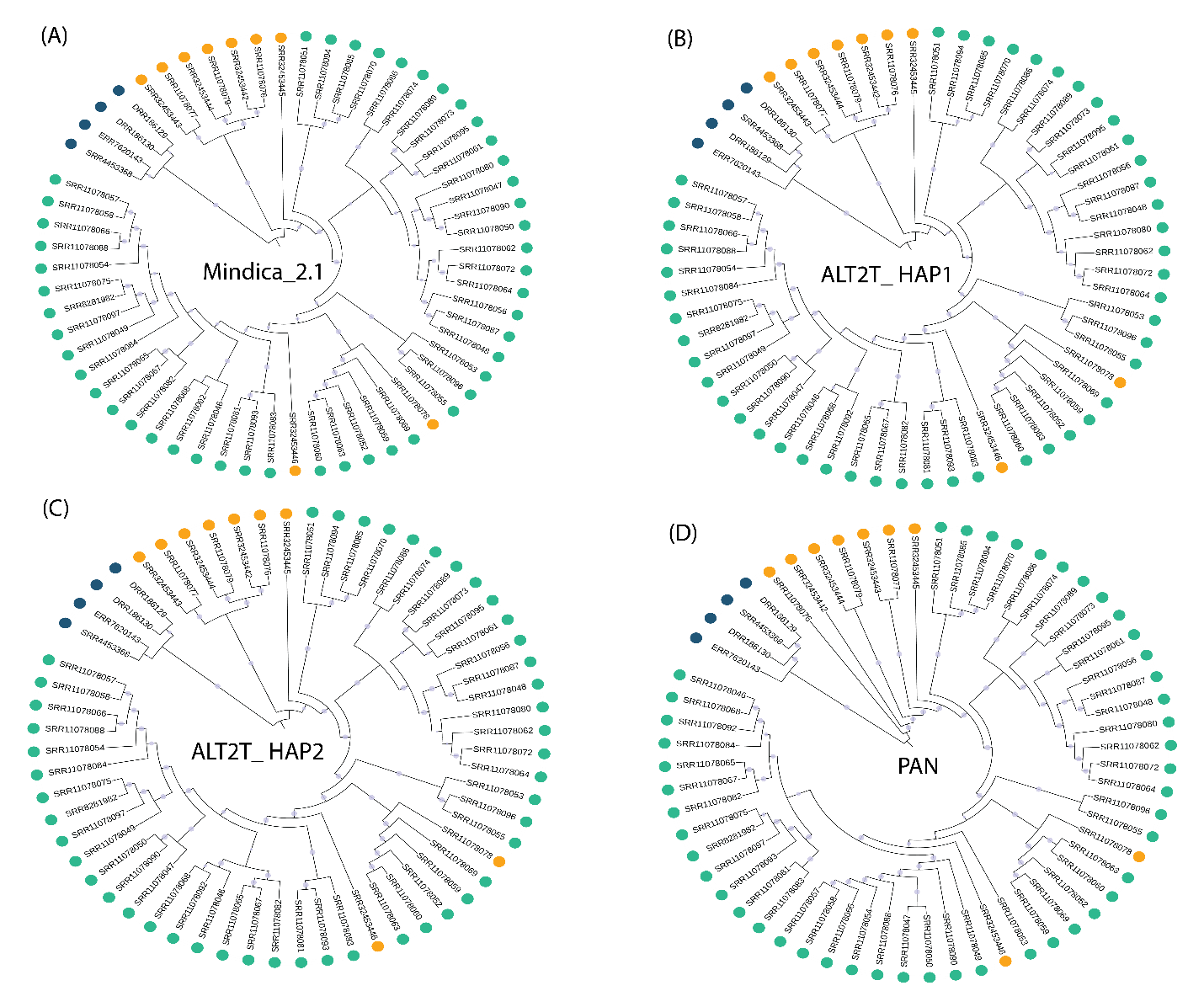


**Figure S7. SV-based phylogenetic tree.** Cultivars, wild types, and the outgroup are represented in green, orange, and blue, respectively. **(A)** Mindica_2.1, **(B)** ALT2T_HAP1, **(C)** ALT2T_HAP2, and **(D)** Pangenome.


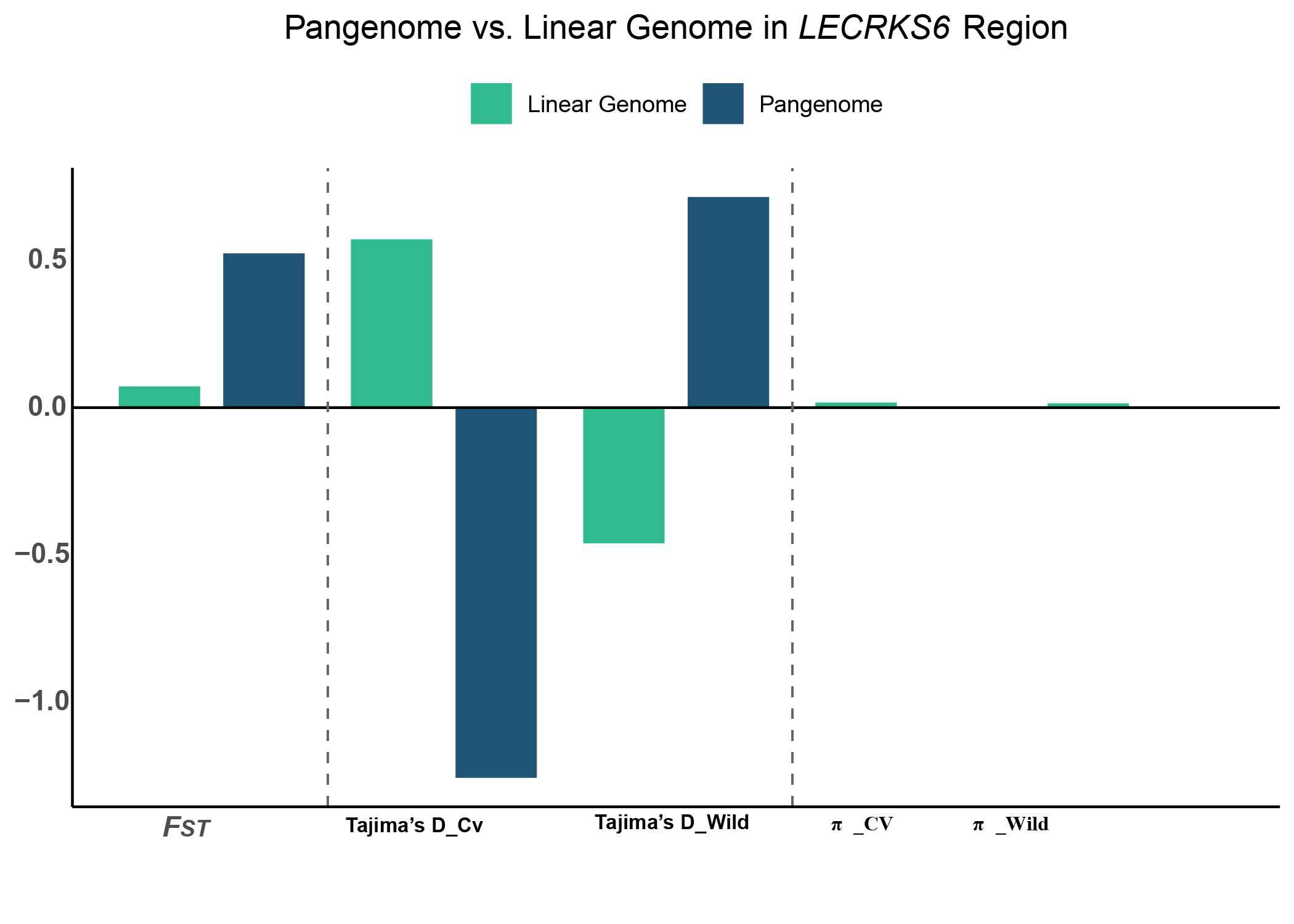


**Figure S8.** Comparison of population genomic metrics between pangenome and linear genome references in the LECRKS6 region. Bar plots represent *F*_ST_, Tajima’s D, and nucleotide diversity (π) across the two references. A solid horizontal line indicates the neutrality threshold (y = 0), while vertical dashed lines separate metric categories.


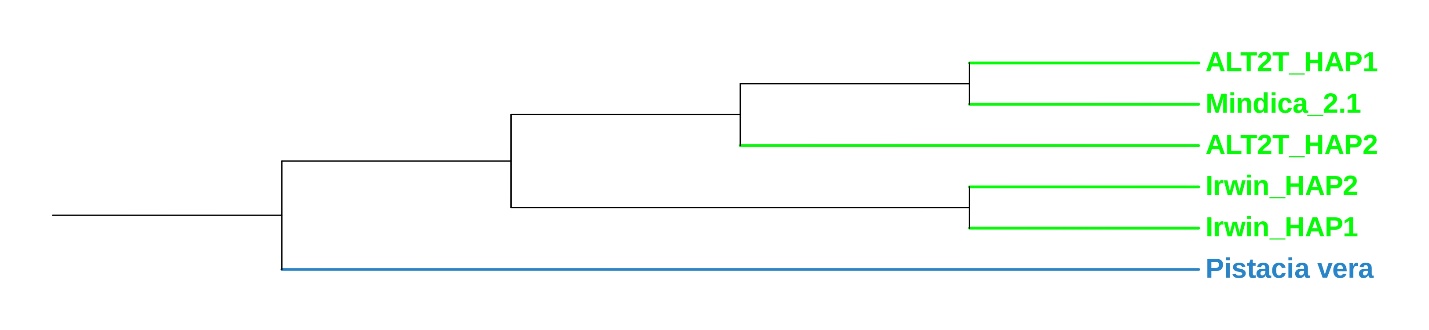


**Figure S9.** Phylogenetic tree of five mango genome assemblies with *Pistacia vera* as the outgroup. The tree was inferred from concatenated alignments of single-copy orthologs using PhyML with the JTT model. Mango assemblies are shown in green; the outgroup is shown in blue.
