# Supplementary material for: Mango pangenome reveals dramatic impacts of reference bias on population genomic analyses
Source: Hortic Res. 2025 Jul 1;12(9):uhaf166. doi: 10.1093/hr/uhaf166 (PMC12344551; doi:10.1093/hr/uhaf166)
Supplement: Web_Material_uhaf166 [file web_material_uhaf166.zip › Supplementary Information_Tables.docx]

**Supplementary Information**

**Table S1.** Start and end positions of centromeres in ALT2T_HAP1 and ALT2T_HAP2.

|  | **CentlER (Mb)** | | **TE visualization in IGV (Mb)** | |
| --- | --- | --- | --- | --- |
| **Chr** | **Start** | **End** | **Start** | **End** |
| Chr1 | 10.8 | 16.5 | 13.27 | 14.04 |
| Chr2 | 6 | 11.7 | 8.44 | 9.27 |
| Chr3 | 17.35 | 18.07 | 15.93 | 16.72 |
| Chr4 | 3.9 | 9.6 | 6.44 | 6.98 |
| Chr5 | 1.94 | 2.64 | 3.79 | 4.24 |
| Chr6 | 9.6 | 15.3 | 12.35 | 12.86 |
| Chr7 | 6.3 | 7.7 | 7.51 | 8.1 |
| Chr8 | 11.48 | 12.186 | 15.95 | 16.52 |
| Chr9 | 3.3 | 9 | 5.9 | 6.29 |
| Chr10 | 10.36 | 11.07 | 11.75 | 12.22 |
| Chr11 | 11.4 | 17.03 | 15.45 | 15.75 |
| Chr12 | 4.48 | 5.29 | 2.12 | 2.82 |
| Chr13 | 2.1 | 7.8 | 4.32 | 4.88 |
| Chr14 | 7.5 | 13.2 | 9.86 | 10.65 |
| Chr15 | 5.62 | 6.24 | 7.79 | 8.14 |
| Chr16 | 7.37 | 8.03 | 7.84 | 8.51 |
| Chr17 | 5.7 | 11.4 | 8.58 | 8.84 |
| Chr18 | 6.37 | 7.2 | 4.47 | 5 |
| Chr19 | 2.7 | 5.7 | 1.56 | 1.71 |
| Chr20 | 5.99 | 6.69 | 4.72 | 5 |

|  | **CentlER (Mb)** | | **TE visualization in IGV (Mb)** | |
| --- | --- | --- | --- | --- |
| **Chr** | **Start** | **End** | **Start** | **End** |
| Chr1 | 10.5 | 16.2 | 13.22 | 13.91 |
| Chr2 | 6.9 | 12.6 | 9.29 | 9.89 |
| Chr3 | 12.9 | 18.6 | 16.22 | 16.62 |
| Chr4 | 3.6 | 9.3 | 6.18 | 6.67 |
| Chr5 | 1.2 | 6.9 | 3.7 | 4.22 |
| Chr6 | 9.9 | 15.6 | 12.51 | 13.02 |
| Chr7 | 8.6 | 9.26 | 6.25 | 6.76 |
| Chr8 | 14.55 | 15.24 | 15.92 | 16.45 |
| Chr9 | 3 | 8.7 | 5.62 | 6.04 |
| Chr10 | 9.9 | 15.6 | 12.51 | 13.06 |
| Chr11 | 12 | 17.32 | 15.81 | 16.29 |
| Chr12 | 2.7 | 5.7 | 2.14 | 2.71 |
| Chr13 | 2.78 | 3.52 | 4.14 | 4.42 |
| Chr14 | 12.59 | 13.3 | 10.18 | 10.92 |
| Chr15 | 6.75 | 7.46 | 7.7 | 8.3 |
| Chr16 | 5.1 | 10.8 | 7.79 | 8.17 |
| Chr17 | 5.7 | 11.4 | 8.58 | 8.99 |
| Chr18 | 2.4 | 8.1 | 4.55 | 5.06 |
| Chr19 | 2.7 | 5.7 | 0.98 | 1.7 |
| Chr20 | 2.1 | 7.8 | 4.63 | 5.03 |

**Table S2.** Comparative genomic analysis of available mango genomes

| **Mango Cv. (Assembly)** | **Tommy Atkins** | **Hong Xiang Ya** | **Alphonso**  **(Mindica_2.1)** | **Irwin** | **Irwin**  **(Hap1)** | **Irwin**  **(Hap2)** | **Irwin**  **(Collapsed)** | **Alphonso**  **(ALT2T_HAP1)** | **Alphonso**  **(ALT2T_HAP2)** |
| --- | --- | --- | --- | --- | --- | --- | --- | --- | --- |
| **Sequencing Technology** | Illumina HiSeq | PacBio CLR+ Illumina | PacBio Sequel II + HiSeq2000 and MiSeq + HiC | PacBio CLR + Illumina + Hi-C | PacBio HiFi | PacBio HiFi | PacBio HiFi | PacBio HiFi  HiC  ONT | PacBio HiFi  HiC  ONT |
| **Assembly method** | DeNovo Magic v. 3.0 | Falcon version 0.3.0 | Canu v. 1.8 | MECAT (version 1.2); Polish (version 1.22) | HiFiasm | HiFiasm | HiFiasm | HiFiasm,  Juicer, 3D-DNA | HiFiasm,  Juicer, 3D-DNA |
| **Genome coverage (x)** | 180 | 388 | 240 | 61.96 | 204 | 204 | 204 | hifi:88.4  ont:149.4 | hifi:88.12  ont:148.88 |
| **Genome size (Mb)** | 374.8 | 371.62 | 392 | 396 | 354.24 | 354.62 | 364.62 | 346.22 | 347.59 |
| **Number of Contigs** | 17,187 | 120 | 420 | 3022 | 4711 | 1515 | 4642 | 397 | 70 |
| **Contig N50 (Mb)** | 0.040 | 4.82 | 3.50 | 1.03 | 13.21 | 15.45 | 14.98 | 17.03 | 17.96 |
| **Number of scaffolds** | 2,565 | - | 252 | 1,305 | 23 | 23 | 23 | 390 | 65 |
| **N50 scaffold (Mb)** | 16.2 | 18.78 | 17.6 | 1.03 | _ | - | - | 18.06 | 19.25 |
| **Telomers** | - | - | - | - | 37 | 37 | 40 | 37 | 36 |
| **BUSCO% (Embryophyta)** | 94.6 | 93.3 | 95.9 | _ | 98.1 | 99 | 99.2 | 99.1 | 99.1 |
| **No. of predicted genes** | 26 616 | 34,529 | 41251 | 36,756 | 34,659 | 33,230 | 35,220 | 41423 | 40794 |
| **Accession**  **number** | [PRJCA005296](https://ngdc.cncb.ac.cn/bioproject/browse/PRJCA005296) | [PRJCA002248](https://ngdc.cncb.ac.cn/bioproject/browse/PRJCA002248) | [PRJNA487154](https://www.ncbi.nlm.nih.gov/bioproject/PRJNA487154/) | CRA004336 | GWHESFJ00000000 | GWHESFK00000000 | GWHEQCT00000000 | This study | This study |

**Table S3.** Details of SNPs and SVs detected in all genomes.

Total number of SNPS

| Mindica_2.1 | 14415958 |
| --- | --- |
| ALT2T_HAP1 | 14770761 |
| ALT2T_HAP2 | 14702268 |
| Pangenome | 14840763 |

Total number of SVs

| Mindica_2.1 | 68393 |
| --- | --- |
| ALT2T_HAP1 | 69889 |
| ALT2T_HAP2 | 69806 |
| Pan | 141169 |

**Table S4.** List of whole genome resequencing samples used.

| SRR11078046 | Guire | Cultivar |
| --- | --- | --- |
| SRR11078047 | Guifei | Cultivar |
| SRR11078048 | Ehokaumn | Cultivar |
| SRR11078049 | Edward | Cultivar |
| SRR11078050 | Yuwen | Cultivar |
| SRR11078051 | Yuanjiang | Cultivar |
| SRR11078052 | Dongfang | Cultivar |
| SRR11078053 | Xiamao | Cultivar |
| SRR11078054 | Tommy | Cultivar |
| SRR11078055 | Tianyang | Cultivar |
| SRR11078056 | Thany Dom | Cultivar |
| SRR11078057 | Smith | Cultivar |
| SRR11078058 | Sensation | Cultivar |
| SRR11078059 | Sanya | Cultivar |
| SRR11078060 | Sannian | Cultivar |
| SRR11078061 | Sanluedoo | Cultivar |
| SRR11078062 | Rad | Cultivar |
| SRR11078063 | Danzhou | Cultivar |
| SRR11078064 | Pat Ban Lad | Cultivar |
| SRR11078065 | Pan Pu Zun | Cultivar |
| SRR11078066 | Palmer | Cultivar |
| SRR11078067 | Pa Lu | Cultivar |
| SRR11078068 | Neelum | Cultivar |
| SRR11078069 | Napo | Cultivar |
| SRR11078070 | Nanxi | Cultivar |
| SRR11078072 | Nam Doc Mai | Cultivar |
| SRR11078073 | Mum Kan Sei | Cultivar |
| SRR11078074 | Chokanan | Cultivar |
| SRR11078075 | Mulgoba | Cultivar |
| SRR11078080 | Mandoum Kao | Cultivar |
| SRR11078081 | Macheso | Cultivar |
| SRR11078082 | Ma Saw Yin | Cultivar |
| SRR11078083 | Laofengzhai | Cultivar |
| SRR11078084 | Kyay Ni | Cultivar |
| SRR11078085 | Banna | Cultivar |
| SRR11078086 | Khiew Swoey | Cultivar |
| SRR11078087 | Khiew Swoey | Cultivar |
| SRR11078088 | Keitt | Cultivar |
| SRR11078089 | Keawsaweu | Cultivar |
| SRR11078090 | JingHwang | Cultivar |
| SRR11078092 | Hongyu | Cultivar |
| SRR11078093 | Hongtao | Cultivar |
| SRR11078094 | Hekou | Cultivar |
| SRR11078095 | Haew | Cultivar |
| SRR11078096 | Baise | Cultivar |
| SRR11078097 | Amrapali | Cultivar |
| SRR8281982 | Alphonso | Cultivar |
| SRR11078076 | *Mangifera sylvatica* | Wild |
| SRR11078077 | *Mangifera persiciforma* | Wild |
| SRR11078078 | *Mangifera odorata* | Wild |
| SRR11078079 | *Mangifera hiemalis* | Wild |
| SRR32453442 | *Mangifera sylvatica Roxb* | Wild |
| SRR32453443 | *Mangifera altissima* | Wild |
| SRR32453444 | *Mangifera odorata* | Wild |
| SRR32453445 | *Mangifera siamensis* | Wild |
| SRR32453446 | *Mangifera ANO* | Wild |
| DRR186129 | *Bouea macrophylla* | Outgroup |
| DRR186130 | *Spondias dulcis* | Outgroup |
| ERR7620143 | *Toxicodendron pubescens* | Outgroup |
| SRR4453368 | *Pistacia vera* | Outgroup |
